# Tools for measuring medical internship experience: a scoping review

**DOI:** 10.1186/s12960-021-00554-7

**Published:** 2021-01-14

**Authors:** Yingxi Zhao, Peris Musitia, Mwanamvua Boga, David Gathara, Catia Nicodemo, Mike English

**Affiliations:** 1 Oxford Centre for Global Health Research, Nuffield Department of Medicine, University of Oxford, S Parks Rd, Oxford, OX1 3SY United Kingdom; 2 KEMRI-Wellcome Trust Research Programme, Nairobi, Kenya; 3 Nuffield Department of Primary Care Health Sciences, University of Oxford, Oxford, United Kingdom

**Keywords:** Intern, House officer, Foundation doctor, Experience, Well-being, Educational environment, Medical training

## Abstract

**Background:**

Appropriate and well-resourced medical internship training is important to ensure psychological health and well-being of doctors in training and also to recruit and retain these doctors. However, most reviews focused on clinical competency of medical interns instead of the non-clinical aspects of training. In this scoping review, we aim to review what tools exist to measure medical internship experience and summarize the major domains assessed.

**Method:**

The authors searched MEDLINE, Embase, PsycINFO, ERIC, and the Cochrane Library for peer-reviewed studies that provided quantitative data on medical intern’s (house officer, foundation year doctor, etc.) internship experience and published between 2000 and 2019. Three reviewers screened studies for eligibility with inclusion criteria. Data including tools used, key themes examined, and psychometric properties within the study population were charted, collated, and summarized. Tools that were used in multiple studies, and tools with internal validity or reliability assessed directed in their intern population were reported.

**Results:**

The authors identified 92 studies that were included in the analysis. The majority of studies were conducted in the US (*n* = 30, 32.6%) and the UK (*n* = 20, 21.7%), and only 14 studies (15.2%) were conducted in low- and middle-income countries. Major themes examined for internship experience included well-being, educational environment, and work condition and environment. For measuring well-being, standardized tools like the Maslach Burnout Inventory (for measuring burnout), Patient Health Questionnaire-9 (depression), General Health Questionnaire-12 or 30 (psychological distress) and Perceived Stress Scale (stress) were used multiple times. For educational environment and work condition and environment, there is a lack of widely used tools for interns that have undergone psychometric testing in this population other than the Postgraduate Hospital Educational Environment Measure, which has been used in four different countries.

**Conclusions:**

There are a large number of tools designed for measuring medical internship experience. International comparability of results from future studies would benefit if tools that have been more widely used are employed in studies on medical interns with further testing of their psychometric properties in different contexts.

## Introduction

In most countries, all doctors must complete a medical internship after completing four to six years of medical education, and before becoming generally licensed and registered with the medical board of that country. This is a structured period where doctors in training transit from supervised learning in medical schools to rapidly assume clinical responsibility under supervision, and it can be challenging [1]. Despite the different terminology from intern, house officer, foundation doctor to resident, they are all under huge pressure: highly demanding working hours, less satisfactory pay and a need for ongoing learning and assessment [2]. Depending on the context, interns may also experience low availability of resources, limited supervision and feedback [3], poor safety climate [4], lack of responsiveness to basic psychological needs that result in rapid burnout and stress [5, 6]. The internship year/years are also the time where these trainees are about to make their first career decisions [7] and they are important in informing opinions about whether they want to continue medicine in that organization and country, and if so which specialty career seems most attractive [8, 9]. Exits after internship have significant financial cost to the host organization and country [10], and evidence from low- and middle-income countries (LMICs) suggests substantial exits and migration to high-income countries immediately after qualification [11–13], causing “brain drain” and huge financial losses as medical education is heavily subsidized in most countries.

While previous systematic reviews have summarized the tools for assessing clinical [14], procedural [15] and psychomotor skills [16] in medical trainees, there has been a lack of focus on understanding what standardized tools are available to measure the experience of internship training. Moreover, there isn’t a common definition of key areas to measure, and the questions in major national trainee surveys differ substantially in each country [17–19]. This limits options for comparison between countries and across time to assess long-term trends or results of interventions/policy changes.

In this scoping review, we aim to fill the literature gap by mapping the existing tools to measure medical internship experience, summarizing the major areas assessed and highlighting the tools used in multiple studies or with psychometric properties assessed directly in the intern population under study. This review may help medical educators, human resource managers and policy makers decide what are the major areas to consider for internship surveys and what are the most appropriate tools available for this purpose.

## Methods

We followed the five steps of Arksey and O’Malley method [20] for scoping review to identify the existing tools to measure medical internship experience. We conducted the review in accordance with PRISMA-ScR standards (see Additional file 1).

Identifying the initial research questions To guide the search strategy and ensure that a broad range of literature was captured, our research question was: “What are the existing tools to measure experience of medical interns?”. We defined medical intern as a physician who has completed their primary academic qualification and within a period of time (usually 1–2 years) typically working in accredited positions in hospital settings, to gain supervised experience (see Box 1). We adopted a wide definition of experience to generate “breadth of coverage” [20].

**Box 1. Different terminology for intern and their case for inclusion**

- **Intern** refers to doctor in training who completed their primary qualification training and spend usually one to two years working in accredited positions in hospital settings. This period is compulsory and may be regulated by the government and professional licensing board. This is the period before they are fully licensed and registered to practise medicine unsupervised. This is the most common term in most countries.
- **House officer,** specifically **pre-registration house officer,** refers to doctor in first year after qualification. This is an official grade term in the UK until 2005 when it was replaced by foundation doctor but is still in use in some other countries, e.g. Nigeria.
- **Foundation doctor** refers to doctor undertaking the UK foundation programme, a 2-year structured programme that bridge between medical school and specialty training. This replaced the house officer grade in 2005.
- **Resident** usually refers to qualified doctor undertaking graduate medical education to obtain a license for a chosen speciality. In the US, the first year of residency is called internship. While there are different requirements in each state and medical programme, licensing usually happen after residency. In our review, we included studies examining residents if they are referring to pre-licensed residents and are not residents from one single specialty.

### Identifying relevant studies

In consultation with an experienced librarian, we conducted a systematic search using MEDLINE, Embase, PsycINFO, Education Resource Information Center and the Cochrane Library to obtain relevant articles. We included quantitative studies published between 2000 and 2019 in English only due to time and resource constraints. We combined keyword terms and phrases related to medical interns (intern, foundation doctor, house officer, resident), tools (survey, questionnaire, assessment, evaluation, scale, index, instrument) and experience (experience, environment, culture, supervision, climate, well-being). To reduce the number of studies to be screened, we also excluded keywords related to qualitative studies, other health workforce cadres, and clinical skills (see Additional file 2 for the search strategies).

### Study selection

We included studies if they (1) examined pre-licensed/registered medical interns (we hand-searched the study population in that country to ensure the study population are pre-licensed and pre-registered); (2) measured or evaluated the non-clinical aspects of the internship experience on an individual level; (3) used a questionnaire or tool (quantitative design); We excluded studies if they (1) examined undergraduate/graduate medical students undergoing clerkships, qualified doctors, specialists, consultants, residents on one single specialty, other health workers or a mix of different population; (2) measured or evaluated the clinical skills (surgical/procedural skills) of medical interns; (3) used a qualitative approach including interviews and focus group discussions.

After deduplication, we imported the citations into Abstrackr for initial title and abstract screening [21]. YZ reviewed all the titles and abstracts to assess eligibility for full-text review, and a random subset of 40% were reviewed by PM and MB together. We used Gwet’s AC1 to assess agreement rate between reviewers, which is an agreement coefficient that perform better than Cohen’s Kappa when prevalence is low [22, 23]. The agreement rate on which study to include between YZ and PM + MB for this subset was high (percent agreement 0.96 [excellent], Cohen’s Kappa 0.47 [moderate], Gwet’s AC1 0.96 [excellent]) therefore we proceeded with the full-text review. YZ reviewed all full texts as primary reviewer and either PM or MB acted as secondary reviewer to determine inclusion (percent agreement 0.81 [excellent], Cohen’s Kappa 0.60 [substantial], Gwet’s AC1 0.65 [substantial]). We resolved disagreements on inclusion at title and abstract stage or at full-text stage by discussion among the three reviewers.

### Data charting and collation

Three reviewers charted data from included articles and entered them into a Microsoft Excel spreadsheet. We extracted the following data items: title, authors, year of publication, country of study, study population category, internship training years, the number of hospitals included, sample size, data collection approach, questionnaire type, full questionnaire availability, any standardized scale or questionnaire used, key terms assessed, psychometric properties. We charted key terms by looking into the method, result, tables/figures, and questionnaire appendix when available, and summarizing the key terms as they were defined or reported in their questionnaire or tool (e.g. burnout, depression, supervision, workload, work hours, etc.).

In the current review, we are also interested in whether the studies reported evidence of internal validity and reliability in their study population. We extracted whether the study (1) provided actual evidence of internal validity (face, content, criterion, concurrent, convergent, discriminant, predictive, construct) and reliability (internal consistency, test–retest or inter-rater) tested within their study population [24], or (2) stated they were previously tested or verified but did not test in their study population, or (3) did not mention them.

### Summarizing and reporting findings

One aim of this review is to identify and summarize the major themes as a way of categorizing the key areas covered by different studies and tools. Major themes assessed by the tools gradually emerged during title and abstract screening and were refined and finalized iteratively during full-text screening and data charting and collation, developed by repeated discussions amongst the three reviewers (YZ, PM and MB) and an additional author (ME). We finalized with three major themes: well-being, educational environment, and work condition and environment.

We further combined and merged key terms extracted in the last step into sub-themes and placed them under each major theme. For example, under well-being we summarized sub-themes including stress, burnout, etc.; under work condition and environment, we merged work hours and workload as one sub-theme. YZ finalized and categorized the sub-themes and whereas there was uncertainty on the allocation of sub-themes, the uncertainties were resolved by discussions among the three reviewers.

We described the major themes and sub-themes assessed, and how many included studies examined each theme and sub-theme. For reporting of the actual tools, as the purpose of our study was to identify more widely used tools and tools with better psychometric evidence, our reporting focused on the tools that were used in multiple studies, and tools with internal validity or reliability assessed directed in their intern population.

## Result

### Search results

**Fig. 1:**
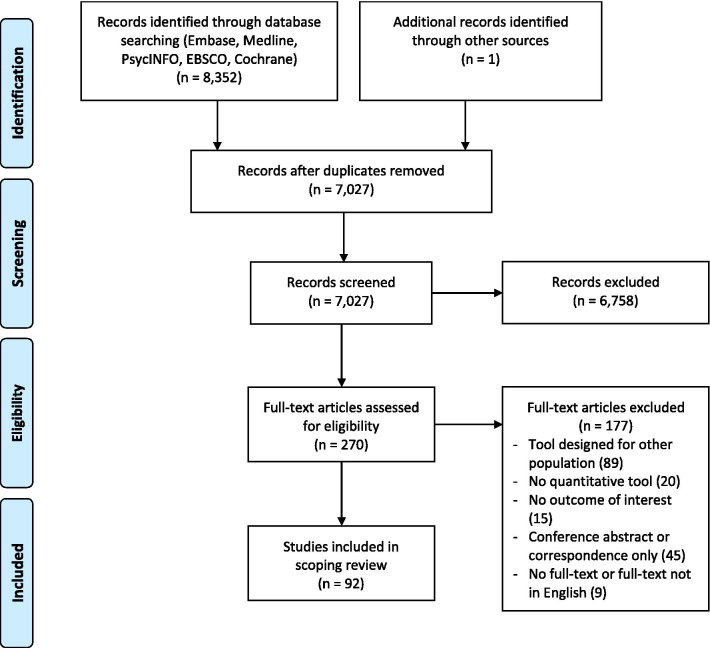
Preferred Reporting Items for Systematic Reviews and Meta‐Analyses flowchart

Figure 1 summarizes the result of the review process. Of 7,027 citations identified after deduplication, 92 met inclusion criteria after the full-text review. The characteristics of included studies are provided in Additional file 3.

### Article overview

Publication dates ranged from 2000 to 2019 with more studies published recently (2000–2004, *n* = 10; 2005–2009, *n* = 22; 2010–2014, *n* = 28; 2015–2019, *n* = 32). The sample size varied from 17 to 91,073, with a median of 172 and interquartile range from 74 to 425. 31 of the included studies specifically examined “interns”, 29 examined “residents”, 12 examined “house officers”, and 10 examined “foundation doctors”. The rest of the studies varied in terms of the study population, e.g. “junior medical officers”, “pre-registration trainees”.

The 92 included studies covered 28 countries with three studies including two countries. The majority of studies (*n* = 78, 84.8%) identified were conducted in high-income countries including US (*n* = 30), UK (*n* = 20), Australia (n = 6), Canada (*n* = 3) while only 14 studies (15.2%) were conducted in LMICs, e.g. Brazil, India, Malawi, Myanmar, Pakistan, South Africa, Sri Lanka.

Most studies used self-reported questionnaires (*n* = 89, 96.7%) and data were mostly collected using paper surveys (*n* = 35, 19 of which were published in or before 2010) or online web surveys (*n* = 31, 25 of which published in or after 2011). One study used a telephone survey, four used mixed-modes (a combination of paper, online and telephone) and 21 did not specify their survey mode.

**Fig. 2:**
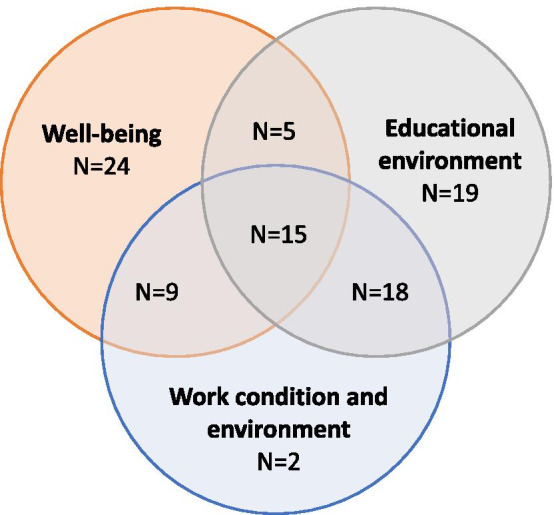
Number of studies focusing on different themes of the internship experience

We summarized the key terms examined into three major themes after data charting, collation and summary: (a) well-being, which mostly examined the physical, mental and social condition of the interns; (b) educational environment, which focused on the educational approach, cultural context and physical location where interns experience learning; and (c) work condition and environment, this referred to the aspects of interns’ terms and condition of employment. Figure 2 shows the number of studies focusing on different themes of the internship experience. Of the 92 studies included, 53 examined well-being, 57 examined educational environment and 44 examined work condition and environment. 47 studies examined more than one theme while 15 studies examined all three themes. Table 1 presents the most commonly assessed sub-themes within these themes, and tools that were used in multiple studies, and tools with internal validity or reliability assessed directed in their intern population.

**Table 1:** Summary of sub-themes assessed and tool used

	Well-being (n = 53)	Educational environment (n = 57)	Work condition and environment (n = 44)
Sub-theme assessed	Stress or psychological distress (*n* = 28) Job satisfaction (*n* = 14) Depression (*n* = 12) Sleep (*n* = 11) Burnout (*n* = 10) Anxiety (*n* = 8) Overall well-being (*n* = 5) Fatigue (*n* = 4) Quality of life (*n* = 3) Wellness (*n* = 2) Empathy (*n* = 1) Coping strategy (*n* = 1) Mood (*n* = 1) Loneliness (*n* = 1) Life satisfaction (*n* = 1)	Supervision (*n* = 26) Support (*n* = 15) Learning or educational environment (*n* = 15) Teaching (*n* = 14) Preparedness (*n* = 14) Teamwork (*n* = 14) Feedback (*n* = 12) Communication (*n* = 11) Induction (*n* = 9) Professionalism (*n* = 8) Handoff (*n* = 6) Career development (*n* = 5) Overall educational experience (*n* = 1)	Workload or work hours (*n* = 35) Safety (*n* = 14) Harassment or bullying (*n* = 11) Food and accommodation (*n* = 10) Pay and remuneration (*n* = 5) Infrastructure (*n* = 3) Work–family conflict (*n* = 2)
Tools used in two or more studies used twice or more, or used once but with actual internal validity or reliability evidence within their study population	Maslach Burnout Inventory (*n* = 6) Patient Health Questionnaire-9 (*n* = 6)* General Health Questionnaire-12 or 30 (*n* = 5) Perceived stress scale (*n* = 4)* Hospital Anxiety and Depression Scale (*n* = 3)* Brief resident wellness profile (*n* = 2)* Connor–Davidson Resilience Scale (*n* = 2)* Cooper Job Stress (*n* = 2)* Copenhagen Burnout Inventory (*n* = 2)* Occupational Stress Indicator (*n* = 2)* Pittsburgh Sleep Quality Index (*n* = 2) UK Medical Career Research Group (*n* = 2) Anxiety about professional future (*n* = 1)* Chalder Fatigue Scale (*n* = 1)* Cohen Perceived Stress Scale (*n* = 1)* Copenhagen Psychosocial Questionnaire (*n* = 1)* Effort–Reward Imbalance (ERI) (*n* = 1)* General stressor questionnaire (*n* = 1)* Job satisfaction scale (*n* = 1)* Medical Outcomes Study Sleep Scale (*n* = 1)* Minnesota Satisfaction Scale (*n* = 1)* Positive and Negative Affect Schedule scales (*n* = 1)* State–Trait Anxiety Inventory (*n* = 1)* State–Trait Depression Scale (*n* = 1)*	UK Medical Career Research Group survey (*n* = 5) Postgraduate Hospital Educational Environment Measure (*n* = 4)* Accreditation Council for Graduate Medical Education Resident Survey (*n* = 2) Junior Doctor Assessment Tool (*n* = 2)* Climate for learning (*n* = 1)* Cognitive Behaviour Survey—Residency (*n* = 1)* Friesen et al. 2008 questionnaire (*n* = 1)* Graduate Medical Education Committee annual survey (*n* = 1)* Handoff Clinical Evaluation Exercise (*n* = 1)* Hannan et al. 2017 questionnaire (*n* = 1)* Learning environment professionalism survey (n = 1)*Lubben Social Network Scale (*n* = 1)* Mentorship effectiveness Scale (*n* = 1)* Modified Resident Questionnaire (*n* = 1)* Reynolds et al. 2019 questionnaire (*n* = 1)*Short Survey of Perceived Organizational Support (*n* = 1)* Safety Attitudes Questionnaire (*n* = 1)* Speaking Up Climates (*n* = 1)* Touchie et al. 2014 questionnaire (*n* = 1)* Work Analysis Instrument for Hospitals (*n* = 1)* Yusoff et al. 2011 questionnaire (*n* = 1)*	Postgraduate Hospital Educational Environment Measure (*n* = 4)* UK Medical Career Research Group survey (*n* = 3) Accreditation Council for Graduate Medical Education Resident Survey (*n* = 2) Cyber Negative Acts Questionnaire (*n* = 1)* Graduate Medical Education Committee annual survey (*n* = 1)* Hannan et al. 2017 questionnaire (*n* = 1)* Modified Resident Questionnaire (*n* = 1)* Psychological Safety Scale (*n* = 1)* Robson et al. 2011 questionnaire (*n* = 1)* Safety Attitudes Questionnaire (*n* = 1)* Speaking Up Climates (*n* = 1)* Work Analysis Instrument for Hospitals (*n* = 1)* Yusoff et al. 2011 questionnaire (*n* = 1)*

n refers to the number of included studies that examined the specific sub-theme or used the tool

* Refers to tools with internal validity or reliability evidence presented in their study population

Regarding psychometric properties, 75 studies did not test for internal validity in their population although 37 of these 75 studies mentioned that the tool used was previously validated in other studies. Of the 17 studies with actual evidence of internal validity assessment in their study population, 12 tested for construct validity, seven for face validity, four for concurrent validity, three for discriminate validity, two for content validity, one for convergent validity, and one for predictive validity. 64 studies did not test for reliability in their population but 15 of these studies reported that the tool was previously tested for reliability. Of the 28 tools that provided reliability evidence in their study population, internal consistency (Cronbach’s alpha) was the most commonly reported reliability assessment (*n* = 27), followed by test–retest reliability (*n* = 3) and inter-rater reliability (*n* = 1).

### Well-being

53 studies examined well-being in interns. The most assessed sub-themes included stress or psychological distress (*n* = 28), job satisfaction (*n* = 14), depression (*n* = 12), sleep (*n* = 11) and burnout (*n* = 10). Other studies also assessed fatigue, quality of life, etc.

The most commonly used tools to assess well-being included the Maslach Burnout Inventory (MBI, *n* = 6) for measuring burnout, the Patient Health Questionnaire (PHQ-9, *n* = 6) for depression, General Health Questionnaire (GHQ-12 or 30, *n* = 5) for psychological distress, Perceived Stress Scale (*n* = 4) for stress, and Hospital Anxiety and Depression Scale (HADS, *n* = 3) for anxiety and depression. Most studies did not report actual psychometric properties in their population, for the commonly used tools such as MBI and GHQ authors reported that they were previously tested for validity and reliability. One study tested reliability for PHQ-9 and PSS in US interns and both showed good reliability (Cronbach’s alpha = 0.74–0.85 for PHQ-9 and 0.82–0.90 for PSS) [25]. Another study tested the reliability of HADS in a UK house officer population and indicated that HADS has low internal reliability (Cronbach’s alpha = 0.53) [26].

The rest of the tools were only used once or twice in the included studies. There was no widely used tool for job satisfaction, and tools used for this sub-theme varied in each study from scales [27, 28] to a single question on job satisfaction [29, 30]. Detailed information on the rest of the tools categorized as measuring well-being and the other two themes are provided in Additional file 4.

### Educational environment

57 studies examined the educational environment of interns. Supervision (*n* = 26), support (*n* = 16), teaching (*n* = 14), preparedness (*n* = 14) and teamwork (*n* = 14) were the top assessed sub-themes. There were also 15 studies that specifically defined learning or educational environment. Only six studies included handoff and five others included career development.

Of the tools most used to assess educational environment, two of them were large-scale surveys administered in the UK and the US, these reports did not provide validity or internal reliability evidence in the paper published. The UK Medical Career Research Group survey was mentioned in five studies [29–33], which covered different sub-themes including support, handoff, induction, supervision, teaching, feedback, preparedness; and the Accreditation Council for Graduate Medical Education Resident Survey (ACGME) was cited in two studies [34, 35] that assessed teamwork, educational experience and handoff. The other two tools that were used more than once were developed as a stand-alone tool/scale: the Postgraduate Hospital Educational Environment Measure (PHEEM) was used in four studies including in UK (where it was originally developed) [36], Australia [37], Greece [38] and Sri Lanka [39]. PHEEM includes three sub-scales including perceptions of role autonomy, teaching and social support, and has 40 questions in total. It asks specific questions on teaching, supervision, support, feedback, teamwork, communication, induction and career development in regard to the educational environment. PHEEM has shown good reliability in the UK and Sri Lanka (Cronbach’s alpha = 0.93 and 0.84, respectively) and face validity and construct validity [36, 39]. The Junior Doctor Assessment Tool was used twice in Australia in which the supervisors assess and rate the communication skills (three questions), professionalism (three questions) and clinical management skills (four questions) of the junior doctor [40, 41]. The tool showed good construct validity and internal consistency (Cronbach’s alpha = 0.88) [40].

### Work condition and environment

Out of 44 studies that assessed work condition and environment of interns, workload or work hours (*n* = 35), safety (*n* = 14), harassment or bullying (*n* = 11), food and accommodation (*n* = 10) were the most examined sub-themes. Workload or work hours were usually assessed through actually asking duty hours [25, 42, 43] or perception of workload [29, 33, 44]. Pay and remuneration and hospital infrastructure (equipment/commodity availability) were less mentioned in the survey.

There was a significant overlap between tools used in this theme and the educational environment theme. For example, four studies used PHEEM which also covered safety, work hours and harassment [36–39]; three used the UK Medical Career Research Group survey [29, 30, 33] which included questions on workload, harassment or bullying, food and accommodation; two used the ACGME Resident Survey which also included questions on safety and workload [34, 35]. Three separate tools were used in two studies to understand safety issues in the internship period including the Safety Attitudes Questionnaire, Speaking Up Climate, and Psychological Safety Scale [45, 46]. In the first study, Speaking Up Climate was used in residents from the US to compare against Safety Attitudes Questionnaire, and had good internal consistency (Cronbach’s alpha = 0.79) and discriminant and concurrent validity [46]. The second validated the Psychological Safety Scale in another US resident population with good internal consistency (Cronbach’s alpha = 0.76) and concurrent validity [45].

## Discussion

This review summarized tools designed to measure medical internship experience. We defined “internship” as the period where doctors in training gain supervised experience working in accredited positions in hospital settings before they are fully licensed and registered to practise unsupervised. We adopted a wide definition of internship experience and summarized the areas examined in 92 articles into three major themes (well-being, educational environment, work condition and environment). We found more tools that have be used in multiple settings for well-being, and less tools for the other two themes.

Medical internship is an important period though less examined or emphasized when compared to medical students or licensed doctors. Failure to account for providing appropriate and well-resourced internship training could lead to challenges in recruiting and retaining these health professionals contributing to the global shortage of physicians, as the internship period appears to be a critical time in career decision-making for most medical graduates [7]. Medical graduates are soon to be registered and licensed, decide on a specialty or even migrate to another country citing reasons including negative experience as an intern [47], dissatisfaction with the health organization [8] and risky working environment [48]. While there has been increasing emphasis on physician burnout and how that threatens quality of care especially patient safety [49, 50], it is largely focused on more senior practitioners. However, burnout is also prevalent among interns and junior doctors [5] including in our included studies [51, 52], and often times interns are at the frontline of patient management especially in LMICs [53].

It should be noted that there wasn’t a common definition of “internship experience” across different studies and the key areas to measure. The questions included for several major national trainee surveys like the UK General Medical Council (GMC) National Training Survey and the ACGME resident/fellow survey also vary significantly. These larger trainee surveys, conducted by the medical councils, are not exclusively for interns and also their primary objective is to monitor and report on the quality of medical education and training therefore they are less focused on trainee wellness and personal development. For example, the GMC National Training Survey did not include any questions related to well-being until 2019 when a burnout inventory was added [17] and the ACGME resident/fellow survey on well-being only covered questions on “instruction on maintaining physical and emotional well-being”, “program instruction in when to seek care regarding fatigue and sleep deprivation; depression; burnout; substance abuse” [19]. Moreover, these surveys are tailored to the regulations of that specific country and therefore might not be easily translated to other countries, limiting options for comparison across countries.

Most studies were conducted in high-income countries, whereas only 14 studies out of 92 were conducted in LMICs. The fact that few studies have been conducted in LMICs reflects the neglect of this issue in countries with the most significant shortage of doctors [54], and where higher proportions of medical students and interns intend to migrate after qualification citing dissatisfaction with their education and training and poor working conditions [11–13]. This not only leads to “brain drain”, but also great economic losses to the LMICs of origin. Some LMICs try and enforce contracts binding interns to a further minimum period of work post-licensure in their country. However, the governments, medical schools and medical councils should also consider methods to reduce ‘push factors’ such as improving conditions for medical students, interns and junior doctors, and optimizing their experience throughout their training and professional career [55].

For well-being, we found that studies commonly examined stress and psychological distress, job satisfaction, depression, sleep, burnout and anxiety. More standardized tools have been in use to measure these sub-themes other than sleep and job satisfaction. Despite widely used across different populations and settings [56–59], tools like MBI and GHQ did not have evidence presented for internal validity and reliability in our included studies. We recommend that these previously validated tools be tested in new contexts and populations when used.

For educational environment, most studies included questions on supervision, teaching, support, but few examined induction, communication, career development which perhaps should be given more emphasis career decisions are commonly made during the internship period [7]. There has been a lack of widely used tools in interns other than PHEEM. A previous systematic review also suggested the use of PHEEM for postgraduate medicine’s educational environment [60]. Most other widely used tools for this theme that we included in our analysis either was tailored to specific country settings (e.g. the ACGME survey [34, 35] and UK Medical Career Research Group survey [29–33]) or only focused on specific sub-themes. Given its greater use, we recommend the further adaptation and use of PHEEM to measure educational environments and enable comparison across settings.

Lastly, for work condition and environment, workload, safety, pay and remuneration were more commonly examined in our included studies. Infrastructure, e.g. equipment and commodity availability were less measured which might reflect the limited number of studies conducted in LMICs. We also did not identify any widely used tools other than PHEEM. While PHEEM does include questions on safety, questions are limited to two on physical safety and no-blame culture [36]. To gain a better picture of this topic we recommend the use of additional tools on safety or safety climate as transforming organizational culture may be critical to improving patient outcomes. Also noteworthy for LMICs there should be questions on pay and remuneration, as together with infrastructure and resource adequacy these factors are commonly cited as reasons for poor internship experience [61].

The list of the tools we reviewed that measured medical internship experience could be of interest to researchers, medical educators, human resource managers and policy makers. Depending on the areas of interest, we recommend the tools that have been widely used in different settings and with sufficient evidence of psychometric properties, for example as listed in Table 2. For surveys that aim to provide a more comprehensive assessment of internship experience, e.g. a national intern survey, we recommend that all three of the major themes we identify be covered. We also strongly suggest continued testing and reporting of internal validity and reliability when using such tools in new contexts and specific populations. In the future, the development of a comprehensive “internship experience tool” might allow comparison across countries and time.

**Table 2:** Selected recommended tools for measuring internship experience

Tool name	Number of	description questions	Sub-themes assessed	Internal validity evidence	Reliability evidence
Maslach Burnout Inventory (MBI)	22	3 components of Burnout (emotional exhaustion, depersonalization, personal achievement) 7 level frequency scale from “never” to “daily”	Burnout	Previously tested	Previously tested
Patient Health Questionnaire-9 (PHQ-9)	9	4 level frequency scale from “not at all” to “nearly every day”	Depression	Previously tested	Internal consistency (Cronbach’s alpha = 0.74–0.85), test–retest [25]
General Health Questionnaire-12 or 30 (GHQ-12 or GHQ-30)	12–30	Short versions of the GHQ-60 questionnaire for psychological distress 4 level frequency scale from “not at all” to “much more than usual”	Psychological distress	Previously tested	Previously tested
Perceived Stress Scale (PSS)	10	4 level frequency scale from “never” to “very often”	Stress	Previously tested	Internal consistency (Cronbach’s alpha = 0.82–90), test–retest [25]
Postgraduate Hospital Educational Environment Measure (PHEEM)	40	3 categories (role autonomy, teaching, social support) 5 level Likert scale from “strongly disagree” to “strongly agree”	Educational environment, supervision, induction, communication, feedback, career development, teamwork, teaching, support, work hour, harassment or bullying, safety, food and accommodation	Face validity, construct validity (three factors) [36] Construct validity (one factor) [39]	Internal consistency (Cronbach’s alpha = 0.93) [36] Internal consistency (Cronbach’s alpha = 0.84) [39]

Several limitations should be considered for this review. To start with, this was a scoping review, therefore we did not aim to systematically assess the quality of included studies. While we reported on the psychometric properties for each study, we only focused on whether they tested for internal reliability and validity within their population. We did not report the tools that were only used once in our included papers and cited that their tools were “previously validated” in other populations. Tracing back to the original reports to check whether the earlier validation work could have improved our report. Second, during our screening, we found different terminology for our study population including house officer, resident, junior doctor, trainee doctor. For those less-commonly used terms, we hand-searched other resources on the requirement for license and registration in the country of study to ensure the population was pre-registered/licensed. However, we may have missed several studies on interns. Additionally, for studies that examined US residents, we excluded those that only investigated one specialty (e.g. surgery residents) considering that their areas of focus might be on the individual specialty instead of general experience. For some studies on US resident we had to assume the population was pre-licensure, however, some included all residents from first to sixth or seventh year [62, 63] and therefore might not be comparable with an intern in other settings. Last but not least, our disaggregation of themes and sub-themes was based on analysing and interpreting the studies’ methods, results, tables and figures. Only half of the included studies provided their actual questionnaire in the paper or appendix and this made the thematic analysis challenging. To address this, each study was examined by two reviewers to extract key terms and a list of sub-themes was formed iteratively and emergently during the data extraction process.

## Conclusion

In conclusion, we identified and described a large number of tools designed for or used to measure medical internship experience. Of these, we recommend future work employs those with more extensive prior use in different settings and with sufficient evidence of adequate psychometric properties. We also recommend future work to adapt and develop a broad internship experience tool that allows comparison across countries and time that can also be used to address the relative lack of research in LMICs.

## Supplementary Information

**Additional file 1:** PRISMA-ScR checklist

**Additional file 2:** Example search strategy in Embase

**Additional file 3:** Characteristics of included studies

**Additional file 4:** Detailed information of tool and questionnaire used in 92 included studies

## Data Availability

All data generated or analysed during this study are included in this published article and its additional files.

## Abbreviations

ACGME: Accreditation Council for Graduate Medical Education Resident Survey
GHQ: General Health Questionnaire
HADS: Hospital Anxiety and Depression Scale
LMICs: Low- and middle-income countries
MBI: Maslach Burnout Inventory
PHQ: Patient Health Questionnaire
PHEEM: Postgraduate Hospital Educational Environment Measure
