# Supplementary material for: Tools for measuring medical internship experience: a scoping review
Source: Hum Resour Health. 2021 Jan 14;19:10. doi: 10.1186/s12960-021-00554-7 (PMC7809831; doi:10.1186/s12960-021-00554-7)
Supplement: Supplementary file 2 — Additional file 2: Example search strategy in Embase [file 12960_2021_554_MOESM2_ESM.docx]

**Additional file 2. Example search strategy in Embase**

Database(s): Embase 1974 to present
Search Strategy:

| **#** | **Searches** | **Results** |
| --- | --- | --- |
| 1 | exp education, medical, graduate/ | 308957 |
| 2 | Clinical Clerkship/ | 13348 |
| 3 | (foundation adj2 doctor*).ti,ab. | 628 |
| 4 | "junior doctor*".ti,ab. | 4809 |
| 5 | "house officer*".ti,ab. | 2222 |
| 6 | (medical and trainee*).ti,ab. | 13796 |
| 7 | (medical and (intern or interns or internship*)).ti,ab. | 5295 |
| 8 | "medical resident*".ti,ab. | 2274 |
| 9 | 1 or 2 or 3 or 4 or 5 or 6 or 7 or 8 | 323067 |
| 10 | exp "Surveys and Questionnaires"/ | 693348 |
| 11 | (questionnaire* or survey*).ti,ab. | 1370946 |
| 12 | (assessment* or evaluation* or scale* or index* or tool* or instrument*).ti,ab. | 5394065 |
| 13 | 10 or 11 or 12 | 6300213 |
| 14 | 9 and 13 | 103586 |
| 15 | (experience* or culture or supervision* or environment* or climate* or "well being" or wellbeing).ti,ab. | 3347914 |
| 16 | Social Environment/ | 32668 |
| 17 | 15 or 16 | 3364439 |
| 18 | 9 and 13 and 17 | 34217 |
| 19 | 18 | 34217 |
| 20 | (cardiolog* or neurolog* or "public health*" or oncolog* or cancerolog* or "infectious disease*" or epidemiolog* or immunolog* or haematolog*or surgery* or surgeon* or surgic* or gastroenterolog* or psychiatr* or psychology* or pediatric* or paediatric* or gynecolog* or gynaecolog* or obstetric* or endocrinolog* or rheumatolog* or nutrition* or urolog* or "internal medicine*" or dent* or veterinar* or pharmac* or nurs* or radiolog* or anaesthe* or anesthe* or "emergency medicine*" or "family practice*" or "family medicine*" or "primary care*" or "general practice*" or nephrolog* or geriatric* or dermatolog* or orthopaedic* or orthopaedic* or osteopath* or ophthalmolog* or veterinar* or patholog* or anatomy*).ti,ab. | 7881702 |
| 21 | ("physician assistant*" or "paramedic*" or "nurse*" or "midwife" or "midwives" or "community health worker*").ti,ab. | 346976 |
| 22 | ("interview*" or "focus group discussion*" or qualitative).ti,ab. | 631891 |
| 23 | qualitative research/ or qualitative research.mp. | 83017 |
| 24 | psychomotor performance/ or motor skills/ | 89823 |
| 25 | exp Surgical Procedures, Operative/ or surgical procedures.mp. | 4717327 |
| 26 | ("surgical" or "procedural").ti,ab. | 1315863 |
| 27 | 20 or 21 or 22 or 23 or 24 or 25 or 26 | 11068379 |
| 28 | 27 | 11068379 |
| 29 | 19 not 28 | 7969 |
