# Supplementary material for: Tools for measuring medical internship experience: a scoping review
Source: Hum Resour Health. 2021 Jan 14;19:10. doi: 10.1186/s12960-021-00554-7 (PMC7809831; doi:10.1186/s12960-021-00554-7)
Supplement: Supplementary file 3 — Additional file 3: Characteristics of included studies [file 12960_2021_554_MOESM3_ESM.docx]

**Additional file 3. Characteristics of included studies**

| Authors | Year of publication | Country of study | Study population category | Number of year post graduation | Hospital included number | Participant sample size | Data collection approach | Questionnaire type | Full questionnaire available | Any specific tool used 1 | Any specific tool used 2 | Any specific tool used 3 | Well-being | Educational environment | Work environment and conditions | Validity | Reliability |
| --- | --- | --- | --- | --- | --- | --- | --- | --- | --- | --- | --- | --- | --- | --- | --- | --- | --- |
| Hinze (1) | 2004 | US | Resident | NA | 116 | 99 | Telephone | Self-reported | Selected questions available |  |  |  |  |  | Harassment | 0 | 0 |
| Galam et al. (2) | 2017 | France | Trainee | 1-2 | NA | 343 | Online | Self-reported | Tools available, other question not available | MBI | Jefferson Physician Empathy Scale | Hospital Anxiety and Depression Scale | Burnout, stress, anxiety, depression, empathy, coping strategy |  |  | Previously validated in other studies | 0 |
| Martinez et al. (3) | 2015 | US | Resident | 1-5 | 6 | 837 | Online | Self-reported | Tools available | Safety Attitudes Questionnaire | Speaking up climates |  |  | Teamwork, professionalism | Safety | Face validity, concurrent validity, discriminate validity, construct validity (CFA) | Internal consistency (cronbach's alpha) |
| Kobayashi (4) | 2006 | US and Japan | Resident | 1-2 | NA | 240 | Paper | Self-reported | Questions available | Operating Team Resource Management Survey |  |  |  | Teamwork, professionalism, communication | Safety | 0 | 0 |
| Baldwin et al. (5) | 2003 | US | Resident | 1-2 | NA | 3604 | Paper | Self-reported | Selected questions available | Modified AMA survey |  |  | Stress, sleep, job satisfaction | Supervision, professionalism | Work hour, harassment | 0 | 0 |
| Mitchell et al. (6) | 2009 | US | Resident | 1-3 | 7 | 424 | Online and paper | Self-reported | Tools available | Cognitive Behaviour Survey - Residency |  |  |  | Learning environment |  | Construct validity | Internal consistency (cronbach's alpha) |
| Sen et al. (7) | 2010 | US | Intern | 1 | 13 | 740 | Online | Self-reported | Tools available | PHQ-9 | Perceived Stress Scale | Sarason Social Support Questionnaire, Connor-Davidson Resilience Scale | Depression, sleep, stress | Support | Work hour | Previously validated in other studies | 0 |
| Chu et al. (8) | 2009 | US | Intern | NA | 1 | 72 | NA | Self-reported | Selected questions available |  |  |  |  | Handoff, supervision |  | 0 | 0 |
| Ross et al. (9) | 2018 | South Africa | Intern | 1-2 | 1 | 41 | Paper | Self-reported | Selected questions available |  |  |  |  | Supervision, teaching | Work hour, infrastructure | 0 | 0 |
| Brant et al. (10) | 2010 | UK | Preregistration house officer | 1 | NA | 36 | NA | Self-reported | Tools available | MBI | Hospital Anxiety and Depression Scale |  | Burnout, anxiety, depression |  |  | Previously validated in other studies | Internal consistency (cronbach's alpha) |
| Heard et al. (11) | 2004 | US | Resident | NA | NA | 427 | Online | Self-reported | Selected questions available |  |  |  |  | Supervision, feedback | Workload | Construct validity, content validity, discriminate validity | Internal consistency (cronbach's alpha) |
| Bolanowski (12) | 2005 | Poland and France | Intern | NA | NA | 986 | Paper | Self-reported | Selected questions available | Anxiety about professional future |  |  | Anxiety |  |  | Construct validity (CFA) | Internal consistency (cronbach's alpha) |
| Han & Maxwell (13) | 2006 | UK | Foundation year | NA | NA | 100 | Paper | Self-reported | Questions available |  |  |  |  | Preparedness |  | 0 | 0 |
| Carr et al. (14) | 2013 | Australia | Junior doctor | 1 | NA | 302 | NA | Supervisor rated | Tools available | Junior Doctor Assessment Tool |  |  |  | Communication, teamwork, professionalism |  | Face validity, construct validity | Internal consistency (cronbach's alpha) |
| Gooneratne et al. (15) | 2008 | Sri Lanka | Intern | NA | 3 | 86 | NA | Self-reported | Tools available | PHEEM |  |  |  | Educational environment, supervision, induction, communication, feedback, career development, teamwork, teaching, support | Work hour, harassment, safety, bullying, food and accommodation | Construct validity (EFA) | Internal consistency (cronbach's alpha) |
| Loftus et al. (16) | 2019 | US and Japan | Resident | NA | NA | 484 | Online | Self-reported | Selected questions available | ACGME Resident Survey |  |  |  | Teamwork, educational experience, handoff | Workload, safety | 0 | 0 |
| Lin et al. (17) | 2018 | Myanmar | House officer | NA | 21 | 159 | Online | Self-reported | Tools available | MBI | Satisfaction with Life Scale | Copenhagen Psychosocial Questionnaire | Burnout, life satisfaction | Support |  | Previously validated in other studies | Previously tested in other studies |
| Hannan et al. (18) | 2017 | Ireland | Intern | NA | 4 | 101 | Online | Self-reported | Tools available | MBI | General Health Questionnaire (GHQ-12) |  | Burnout, psychological distress | Support, preparedness, teamwork | Workload | Face validity, construct validity | Previously tested in other studies |
| Calcides et al. (19) | 2019 | Brazil | Intern | NA | 1 | 184 | Paper | Self-reported | Tools available | MBI |  |  | Burnout |  |  | Previously validated in other studies | Previously tested in other studies |
| Li et al. (20) | 2013 | Germany | Resident | NA | NA | 417 | Paper | Self-reported | Tools available | State-Trait Depression Scale | Effort–Reward Imbalance (ERI) |  | Depression, stress |  |  | Previously validated in other studies | Internal consistency (cronbach's alpha) |
| Vinothkumar et al. (21) | 2016 | India | Intern | NA | NA | 120 | NA | Self-reported | Tools available | Perceived stress scale | Occupational stress indicator-2 | Job satisfaction scale | Stress, job satisfaction |  |  | Not clear what type of validity | Internal consistency (cronbach's alpha) |
| Talih et al. (22) | 2015 | Lebanon | Resident | NA | NA | 118 | Online | Self-reported | Tools available | PHQ-9 | Burnout measure (BM) | Generalized anxiety disorder-7 (GAD-7) | Burnout, stress, depression |  |  | Previously validated in other studies | Previously tested in other studies |
| Carr et al. (23) | 2014 | Australia | Junior doctor (PGY1) | 1 | NA | 237 | NA | Supervisor rated | Tools available | Junior Doctor Assessment Tool |  |  |  | Communication, professionalism, teamwork |  | Previously validated in other studies | Previously tested in other studies |
| Roff et al. (24) | 2005 | UK | Preregistration house officer | 1 | 88 | 109 | Paper | Self-reported | Tools available | PHEEM |  |  |  | Educational environment, supervision, induction, communication, feedback, career development, teamwork, teaching, support | Work hour, harassment, safety, bullying, food and accommodation | Face validity | Internal consistency (cronbach's alpha) |
| Robson et al. (25) | 2011 | UK | Foundation year | 1-2 | 3 | 69 | Online | Self-reported | Tools available |  |  |  |  |  | Safety | Content validity, construct validity | 0 |
| Goldacre et al. (26) | 2003 | UK | Preregistration house officer | 1 | NA | 3074 | Paper | Self-reported | Selected questions available |  |  |  | Job satisfaction | Support, handoff, induction, supervision, teaching, feedback | Workload, food and accommodation | 0 | 0 |
| Bleakley & Brennan (27) | 2011 | UK | Foundation year | 1 | NA | 146 | Online and paper | Self-reported | Questions available | Modified Manchester questionnaire |  |  |  | Preparedness |  | 0 | 0 |
| Linklater (28) | 2010 | UK | Foundation year | 1 | 1 | 74 | Paper | Self-reported | Questions available |  |  |  |  | Support, preparedness |  | 0 | 0 |
| Mohammadi et al. (29) | 2019 | Iran | Intern | 1 | 1 | 17 | NA | Self-reported | Tools available | Spielberger's Situational Anxiety Inventory |  |  | Anxiety |  |  | Previously validated in other studies | Previously tested in other studies |
| Kalmbach et al. (30) | 2018 | US | Intern | 1 | 1 | 33 | Online and telephone | Self-reported | Questions available |  |  |  | Sleep, mood |  |  | 0 | 0 |
| Mayer (31) | 2017 | US | Intern | 1 | NA | 74 | Online | Self-reported | Tools available | PHQ-9 | Perceived stress scale |  | Depression, stress |  | Work hours | 0 | Internal consistency (cronbach's alpha), test-retest |
| Farley et al. (32) | 2015 | UK | Foundation year | 1-2 | 8 | 158 | Online | Self-reported | Selected questions available | Cyber Negative Acts Questionnaire | Positive and Negative Affect Schedule (PANAS) scales |  | Wellbeing |  | Bullying | Previously validated in other studies | Internal consistency (cronbach's alpha) |
| Mataya et al. (33) | 2015 | Malawi | Intern | 1-1.5 | 2 | 61 | Paper | Self-reported | Selected questions available |  |  |  | Job satisfaction, quality of life | Learning environment, supervision, career development | Work hours, pay and remuneration | 0 | 0 |
| Friesen et al. (34) | 2008 | US | Intern | 1 | NA | 66 | Paper | Self-reported | Tools available | Cohen Perceived Stress Scale | Chalder Fatigue Scale | Medical Outcomes Study (MOS) Sleep Scale | Stress, fatigue, sleep | Teamwork | Work hours | Previously validated in other studies | Internal consistency (cronbach's alpha) |
| Auret et al. (35) | 2013 | Australia | Intern | 1 | 5 | 74 | Online | Self-reported | Tools available | PHEEM |  |  |  | Educational environment, supervision, induction, communication, feedback, career development, teamwork, teaching, support | Work hour, harassment, safety, bullying, food and accommodation | Previously validated in other studies | Previously tested in other studies |
| Miles et al. (36) | 2015 | UK | Foundation year | 1 | 13 | 192 | Paper | Self-reported | Selected questions available |  |  |  |  | Induction |  | 0 | 0 |
| O'Donnell et al. (37) | 2012 | UK | Foundation year | 2 | NA | 147 | NA | Self-reported | Tools available | State-Trait Anxiety Inventory |  |  | Anxiety |  |  | Previously validated in other studies | Test-retest |
| Rogers E. et al. (38) | 2016 | Canada | Resident | 1-5 | NA | 198 | Online | Self-reported | Tools available | Copenhagen Burnout Inventory | Lubben Social Network Scale |  | Burnout, loneliness | Support |  | Concurrent validity | Internal consistency (cronbach's alpha) |
| Bruce et al. (39) | 2003 | Australia | Intern | 1 | 2 | 302 | NA | Self-reported | Tools available | GHQ |  |  | Psychological distress, wellbeing |  |  | Previously validated in other studies | 0 |
| McKavanagh et al. (40) | 2012 | UK | Foundation year | 1-2 | NA | 215 | Online | Self-reported | Questions available |  |  |  |  | Supervision, feedback |  | 0 | 0 |
| Shapiro et al.(41) | 2019 | US | Trainee | NA | NA | 258 | NA | Self-reported | Tools available | PHQ-9 |  |  | Wellbeing |  |  | Previously validated in other studies | Previously tested in other studies |
| Arora et al. (42) | 2013 | US | Intern | 1 | 2 | 31 | Online | Peer rated | Selected questions available | the Handoff Clinical Evaluation Exercise |  |  |  | Professionalism, communication, feedback, handoff | Workload | Previously validated in other studies | Interrater reliability, internal consistency (cronbach's alpha) |
| Arora et al. (43) | 2010 | US | Intern | 1 | 3 | 299 | Paper | Self-reported | Selected questions available |  |  |  | Fatigue | Supervision | Work hours | 0 | 0 |
| Gruppen et al. (44) | 2015 | US | Resident | NA | 20 | 798 | Online | Self-reported | Selected questions available | Modified Resident Questionnaire |  |  |  | Learning environment | Workload | 0 | Internal consistency (cronbach's alpha) |
| Rovik et al. (45) | 2007 | Norway | Intern | 1 | NA | 402 | Paper | Self-reported | Tools available | Cooper Job Stress |  |  | Stress |  | Work hours | Construct validity, face validity | Internal consistency (cronbach's alpha) |
| Chandramouleeswaranet al. (46) | 2014 | India | Intern | 1 | 1 | 93 | Paper | Self-reported | Tools available | Health Consultant’s Job Stress and Satisfaction questionnaire |  |  | Stress, satisfaction |  |  | 0 | 0 |
| Henning et al. (47) | 2013 | New Zealand | Junior doctor in their first year | 1 | 1 | 17 | NA | Self-reported | Tools available | Depression, Anxiety and Stress Scales | Eurohis quality of life | Copenhagen Burnout Inventory (CBI) | Depression, stress, burnout, quality of life |  |  | Previously validated in other studies | Previously tested in other studies |
| Byszewski et al. (48) | 2017 | Canada | Resident | NA | 2 | 187 | Online | Self-reported | Tools available | Learning environment professionalism survey |  |  |  | Learning environment |  | Previously validated in other studies | Internal consistency (cronbach's alpha), test-retest |
| Keim et al. (49) | 2006 | US | Resident | 1-3 | 1 | 34 | Online | Self-reported | Tools available | Brief resident wellness profile | Stress profile | Positive states of mind | Wellness, satisfaction, stress |  | Work hour | Convergent validity, discriminant validity, concurrent validity, face validity | Internal consistency (cronbach's alpha) |
| Hoppe et al. (50) | 2009 | Sweden | Intern | 1-2 | NA | 69 | Online | Self-reported | Selected questions available |  |  |  |  | Preparedness, feedback, supervision, communication, training |  | Previously validated in other studies | 0 |
| Han et al. (51) | 2014 | South Korea | Intern | 1 | 1 | 61 | Paper | Self-reported | Selected questions available | Mentorship effectiveness scale | Minnesota Satisfaction Questionnaire |  | Job satisfaction | Supervision |  | Previously validated in other studies | Internal consistency (cronbach's alpha) |
| Bu et al. (52) | 2019 | UK | Foundation year | 1-2 | 1 | 20 | Paper | Self-reported | Selected questions available |  |  |  | Stress, well-being |  |  | 0 | 0 |
| Cave et al. (53) | 2007 | UK | Junior doctor in their first year | 1 | NA | 4874 in 2005, 5330 in 2000 | Paper | Self-reported | Selected questions available |  |  |  |  | Preparedness |  | 0 | 0 |
| Kazmi et al. (54) | 2008 | Pakistan | House officer | NA | 1 | 55 | NA | Self-reported | Not available |  |  |  | Stress | Communication, supervision | Workload | 0 | 0 |
| Hassan et al. (55) | 2014 | Pakistan | House officer | NA | 1 | 269 | NA | Self-reported | Tools available | Perceived stress scale |  |  | Stress |  |  | Previously validated in other studies | Previously tested in other studies |
| Cedfeldt et al. (56) | 2010 | US | Resident | 1-4+ | 1 | 445 | Online | Self-reported | Questions available |  |  |  | Stress, job satisfaction, wellbeing, sleep |  |  | Previously validated in other studies | 0 |
| Kleim et al. (57) | 2014 | Switzerland | Intern | NA | NA | 47 | Online | Self-reported | Tools available | PHQ-9 | Connor-Davidson-Resilience Scale |  | Depression, stress |  |  | Previously validated in other studies | Internal consistency (cronbach's alpha) |
| Abuhusain et al. (58) | 2009 | Ireland | Intern | NA | NA | 99 | Paper | Self-reported | Not available |  |  |  |  | Preparedness, induction, learning environment |  | 0 | 0 |
| Kelly et al. (59) | 2011 | Australia | Intern | 1 | 1 | 52 | Online | Self-reported | Selected questions available |  |  |  |  | Preparedness |  | 0 | 0 |
| Goldacre et al. (60) | 2003 | UK | Preregistration house officer | 1 | NA | 3446 | Paper | Self-reported | Selected questions available |  |  |  |  | Preparedness |  | 0 | 0 |
| Charalambous et al. (61) | 2007 | Cyprus | Preregistration trainee | NA | NA | 79 | Paper | Self-reported | Questions available | A 60-item instrument developed by Bellini |  |  |  | Feedback, learning environment, supervision | Workload, infrastructure, harassment, pay and renumeration, food and accommodation | 0 | 0 |
| Lau et al. (62) | 2017 | Australia | Junor medical officer | NA | NA | 1085 | Online | Self-reported | Tool and selected questions available | Kessler Psychological Distress Scale |  |  | Anxiety and depression |  | Work hours | 0 | 0 |
| Lin et al. (63) | 2013 | Taiwan/China | Intern | 1 | 1 | 74 | Paper | Self-reported | Tools available | Beck depression and anxiety inventories |  |  | Anxiety, depression |  |  | Previously validated in other studies | Previously tested in other studies |
| Rodger et al. (64) | 2002 | UK | Preregistration house officer | NA | 3 | 66 | Paper | Self-reported | Tool and selected questions available | GHQ-12 |  |  | Psychological distress, job satisfaction | Support, supervision | Workload | Previously validated in other studies | 0 |
| Al Sultan et al. (65) | 2002 | Saudi Arbia | Intern | 1 | NA | 101 | NA | Self-reported | Selected questions available | Modified questionnaire by Buckey and Harasym |  |  | Stress | Supervision, feedback, communication | Work hours, food and accommodation, harassment | 0 | 0 |
| Appelbaum et al. (66) | 2018 | US | Resident | NA | NA | 322 and 496 | Paper | Self-reported | Tools available | Short Survey of Perceived Organizational Support | Psychological Safety Scale |  |  | Support, learning environment | Safety | Concurrent validity, construct validity | Internal consistency (cronbach's alpha) |
| Newbury-Birch et al. (67) | 2001 | UK | Preregistration house officer | NA | 18 | 109 | NA | Self-reported | Tools available | Occupational Stress Indicator | GHQ-30 | Hospital Anxiety and Depression Scale | Anxiety, depression, psychological distress, job satisfaction |  |  | 0 | 0 |
| Choi et al. (68) | 2017 | US | Resident | 1-4+ | 1 | 2200 | Online | Self-reported | Questions available | MBI |  |  | Burnout, stress, job satisfaction, sleep |  | Work hours | Previously validated in other studies | 0 |
| Reynolds et al. (69) | 2019 | US | Resident | NA | NA | 365 | Online | Self-reported | Questions available |  |  |  |  | Professionalism |  | 0 | Internal consistency (cronbach's alpha) |
| Holt et al. (70) | 2010 | US | Resident | NA | NA | 91,073 | Online | Self-reported | Questions available | ACGME Resident Survey |  |  |  | Supervision, feedback, learning environment | Work hours, safety | Predictive validity, construct validity | Internal consistency (cronbach's alpha) |
| Baldwin et al. (71) | 2018 | US | Resident | 2-3 | 16 | 759 | Online | Self-reported | Selected questions available |  |  |  |  | Supervision |  | 0 | 0 |
| Baldwin & Daugherty (72) | 2004 | US | Resident | 1-2 | NA | 3604 | Paper | Self-reported | Selected questions available |  |  |  | Stress, sleep | Supervision, overall learning, professionalism | Work hours, harassment | Previously validated in other studies | 0 |
| Min et al. (73) | 2015 | US | Resident | 1 | 1 | 69 | NA | Self-reported | Selected questions available | Brief Resident Wellness Profile | Pittsburgh Sleep Quality Index |  | Wellness, sleep disturbance |  |  | Previously validated in other studies | Previously tested in other studies |
| Zebrowski et al. (74) | 2018 | US | Resident | 1 | NA | 281 | Paper | Self-reported | Tool and selected questions available | Pittsburgh Sleep Quality Index | Epworth Sleepiness Scale |  | Sleep |  |  | Previously validated in other studies | Previously tested in other studies |
| Yusoff et al. (75) | 2011 | Malaysia | House officer | NA | 1 | 42 | NA | Self-reported | Tools available | GHQ-12 | General stressor questionnaire | Brief COPE | Psychological distress | Teamwork, supervision, support | Workload, pay, work-family conflict | Previously validated in other studies | Internal consistency (cronbach's alpha) |
| Sangi-Haghpeykar et al. (76) | 2009 | US | Resident | 1-5 | 1 | 275 | Paper | Self-reported | Tools available | Quality of life inventory | Stress inventory |  | Quality of life, stress |  |  | Previously validated in other studies | Previously tested in other studies |
| Kashner et al. (77) | 2010 | US | Resident | 1-7 | NA | 18323 | NA | Self-reported | Questions available |  |  |  | Stress, fatigue | Supervision, learning environment, support, teamwork, teaching | Work hours, infrastructure, safety, food and accommodation | 0 | 0 |
| Touchie et al. (78) | 2014 | Canada | Resident | 1 | NA | 48 | Online | Self-reported | Selected questions available |  |  |  |  | Supervision |  | Face validity | 0 |
| Jagsi et al. (79) | 2008 | US | Resident | 1-6+ | 2 | 1498 | Paper and online | Self-reported | Questions available |  |  |  | Fatigue |  | Work hours, safety | 0 | 0 |
| Marek et al. (80) | 2019 | US | Resident | NA | 1 | 28 | NA | Self-reported | Selected questions available |  |  |  | Burnout, sleep |  |  | Previously validated in other studies | 0 |
| Goldacre et al. (82) | 2008 | UK | Preregistration house officer | 1 | NA | 6243 | Paper | Self-reported | Selected questions available |  |  |  |  | Supervision, support, preparedness | Work hours, remuneration, food and accommodation | 0 | 0 |
| Choi et al. (83) | 2006 | US | Resident | NA | NA | 450 | NA | Self-reported | Selected questions available |  |  |  | Job satisfaction |  | Work hours | Previously validated in other studies | 0 |
| Degen et al. (84) | 2014 | Germany | Resident | 2-3 | NA | 557 | Paper | Self-reported | Selected questions available | Work Analysis Instrument for Hospitals |  |  |  | Teaching, support | Workload | Previously validated in other studies | Internal consistency (cronbach's alpha) |
| Burford et al. (85) | 2014 | UK | Foundation year | 1 | NA | 356 | Paper | Self-reported | Selected questions available |  |  |  |  | Preparedness, overall learning |  | 0 | 0 |
| Anastasiadis et al. (86) | 2018 | Greece | Trainee | NA | 1 | 104 | NA | Self-reported | Tools available | Social Capital Questionnaire (SCQ-G) | Sources of Stress Scale (SSS) | PHEEM | Stress | Educational environment, supervision, induction, communication, feedback, career development, teamwork, teaching, support | Work hour, harassment, safety, bullying, food and accommodation | Previously validated in other studies | 0 |
| Tyssen et al. (87) | 2005 | Norway | Intern | 1 | NA | 371 | Paper | Self-reported | Tool and selected questions available | Cooper's Job stress questionnaire | Climate for learning |  | Stress, sleep | Learning environment, support |  | Construct validity | Internal consistency (cronbach's alpha) |
| Bola et al. (88) | 2015 | South Africa | Intern | 2 | 24 | 90 | Online | Self-reported | Selected questions available |  |  |  |  | Supervision, teaching, handoff, induction | Safety, workload | Previously validated in other studies | 0 |
| Doran et al. (89) | 2007 | UK | Preregistration house officer | 1 | 5 | 149 | Paper | Self-reported | Questions available |  |  |  |  | Preparedness, supervision | Work load | 0 | 0 |
| Lambert et al. (90) | 2013 | UK | Preregistration house officer | 1 | NA | 14329 | Paper | Self-reported | Selected questions available |  |  |  | Job satisfaction | Teaching | Workload | 0 | 0 |
| Guille et al. (90) | 2017 | US | Intern | 1 | 34 | 3121 | Online | Self-reported | Tools available | Work and Family Conflict Scale | PHQ-9 |  | Depression |  | Work-family conflict | Previously validated in other studies | Previously tested in other studies |
| Finucane & O’Dowd [91] | 2005 | Ireland | Intern | 1 | NA | 300 | Paper | Self-reported | Questions available |  |  |  |  | Teaching, feedback, preparedness | Accomodation, pay, harassment, workload | 0 | 0 |
| Swaid et al [92] | 2017 | Saudi Arabia | Intern | 1 | 1 | 71 | Online | Self-reported | Selected questions available |  |  |  | Job satisfaction | Induction, preparedness, teaching |  | 0 | 0 |

**Reference**

1. Hinze SW. ‘Am I Being Over-Sensitive?’ Women’s Experience of Sexual Harassment During Medical Training. Health Interdiscip J Soc Study Health Illn Med. 2004;8:101–27.

2. Galam E, Vauloup Soupault C, Bunge L, Buffel du Vaure C, Boujut E, Jaury P. ‘Intern life’: a longitudinal study of burnout, empathy, and coping strategies used by French GPs in training. BJGP Open. 2017;1:bjgpopen17X100773.

3. Martinez W, Etchegaray JM, Thomas EJ, Hickson GB, Lehmann LS, Schleyer AM, et al. ‘Speaking up’ about patient safety concerns and unprofessional behaviour among residents: validation of two scales. BMJ Qual Saf. 2015;24:671–80.

4. Kobayashi H. A cross-cultural survey of residents’ perceived barriers in questioning/challenging authority. Qual Saf Health Care. 2006;15:277–83.

5. Baldwin DC, Daugherty SR, Tsai R, Scotti MJ. A national survey of residents’ self-reported work hours: thinking beyond specialty. Acad Med J Assoc Am Med Coll. 2003;78:1154–63.

6. Mitchell R, Regan-Smith M, Fisher MA, Knox I, Lambert DR. A New Measure of the Cognitive, Metacognitive, and Experiential Aspects of Residents’ Learning: Acad Med. 2009;84:918–26.

7. Sen S, Kranzler HR, Krystal JH, Speller H, Chan G, Gelernter J, et al. A Prospective Cohort Study Investigating Factors Associated With Depression During Medical Internship. Arch Gen Psychiatry. 2010;67:557.

8. Chu ES, Reid M, Schulz T, Burden M, Mancini D, Ambardekar AV, et al. A Structured Handoff Program for Interns: Acad Med. 2009;84:347–52.

9. Ross A, Naidoo S (Cyril), Dlamini S. An evaluation of the medical internship programme at King Edward VIII hospital, South Africa in 2016. South Afr Fam Pract. 2018;60:187–91.

10. Brant H, Wetherell MA, Lightman S, Crown A, Vedhara K. An exploration into physiological and self-report measures of stress in pre-registration doctors at the beginning and end of a clinical rotation. Stress. 2010;13:155–62.

11. Heard JK, O’Sullivan P, Smith CE, Harper RA, Schexnayder SM. An Institutional System to Monitor and Improve the Quality of Residency Education: Acad Med. 2004;79:858–64.

12. Bolanowski W. Anxiety about professional future among young doctors. Int J Occup Med Environ Health. 2005;18:367–74.

13. Han W, Maxwell S. Are Medical Students Adequately Trained to Prescribe at the Point of Graduation? Views of First Year Foundation Doctors. Scott Med J. 2006;51:27–32.

14. Carr SE, Celenza A, Lake F. Assessment of Junior Doctor performance: a validation study. BMC Med Educ. 2013;13:129.

15. Gooneratne IK, Munasinghe SR, Siriwardena C, Olupeliyawa AM, Karunathilake I. Assessment of psychometric properties of a modified PHEEM questionnaire. Ann Acad Med Singapore. 2008;37:993–7.

16. Loftus TJ, Hall DJ, Malaty JZ, Kuruppacherry SB, Sarosi GA, Shaw CM, et al. Associations Between National Board Exam Performance and Residency Program Emphasis on Patient Safety and Interprofessional Teamwork. Acad Psychiatry. 2019;43:581–4.

17. Lin KS, Zaw T, Oo WM, Soe PP. Burnout among house officers in Myanmar: A cross-sectional study. Ann Med Surg. 2018;33:7–12.

18. Hannan E, Breslin N, Doherty E, McGreal M, Moneley D, Offiah G. Burnout and stress amongst interns in Irish hospitals: contributing factors and potential solutions. Ir J Med Sci 1971 -. 2018;187:301–7.

19. Calcides DAP, Didou R da N, Melo EV de, Oliva-Costa EF de. Burnout Syndrome in medical internship students and its prevention with Balint Group. Rev Assoc Médica Bras. 2019;65:1362–7.

20. Li J, Weigl M, Glaser J, Petru R, Siegrist J, Angerer P. Changes in psychosocial work environment and depressive symptoms: A prospective study in junior physicians: Changes in ERI and Depressive Symptoms. Am J Ind Med. 2013;56:1414–22.

21. Vinothkumar M, Arathi A, Joseph M, Nayana P, Jishma Ej, Sahana U. Coping, perceived stress, and job satisfaction among medical interns: The mediating effect of mindfulness. Ind Psychiatry J. 2016;25:195.

22. Talih F, Warakian R, Ajaltouni J, Shehab AAS, Tamim H. Correlates of Depression and Burnout Among Residents in a Lebanese Academic Medical Center: a Cross-Sectional Study. Acad Psychiatry. 2016;40:38–45.

23. Carr SE, Celenza T, Lake FR. Descriptive analysis of junior doctor assessment in the first postgraduate year. Med Teach. 2014;36:983–90.

24. Roff S, McAleer S, Skinner A. Development and validation of an instrument to measure the postgraduate clinical learning and teaching educational environment for hospital-based junior doctors in the UK. Med Teach. 2005;27:326–31.

25. Robson J, de Wet C, McKay J, Bowie P. Do we know what foundation year doctors think about patient safety incident reporting? Development of a web based tool to assess attitude and knowledge. Postgrad Med J. 2011;87:750–6.

26. Goldacre MJ, Davidson JM, Lambert TW. Doctors’ views of their first year of medical work and postgraduate training in the UK: questionnaire surveys. Med Educ. 2003;37:802–8.

27. Bleakley A, Brennan N. Does undergraduate curriculum design make a difference to readiness to practice as a junior doctor? Med Teach. 2011;33:459–67.

28. Linklater G. Educational needs of foundation doctors caring for dying patients. J R Coll Physicians Edinb. 2010;40:13–8.

29. Mohammadi G, Tourdeh M, Ebrahimian A. Effect of simulation-based training method on the psychological health promotion in operating room students during the educational internship. J Educ Health Promot. 2019;8:172.

30. Kalmbach DA, Fang Y, Arnedt JT, Cochran AL, Deldin PJ, Kaplin AI, et al. Effects of Sleep, Physical Activity, and Shift Work on Daily Mood: a Prospective Mobile Monitoring Study of Medical Interns. J Gen Intern Med. 2018;33:914–20.

31. Mayer S. Examining the Relationships Between Chronic Stress, HPA Axis Activity, and Depression in a Prospective and Longitudinal Study of Medical Internship [Internet]. University of Michigan; 2017. Available from: https://deepblue.lib.umich.edu/handle/2027.42/137074

32. Farley S, Coyne I, Sprigg C, Axtell C, Subramanian G. Exploring the impact of workplace cyberbullying on trainee doctors. Med Educ. 2015;49:436–43.

33. Mataya AA, Macuvele ME, Gwitima T, Muula AS. Factors affecting job satisfaction and commitment among medical interns in Malawi: a cross-sectional study. Pan Afr Med J [Internet]. 2015 [cited 2020 Sep 9];21. Available from: http://www.panafrican-med-journal.com/content/article/21/174/full/

34. Friesen LD, Vidyarthi AR, Baron RB, Katz PP. Factors associated with intern fatigue. J Gen Intern Med. 2008;23:1981–6.

35. Auret K, Skinner L, Sinclair C, Evans S. Formal assessment of the educational environment experienced by interns placed in rural hospitals in Western Australia. :12.

36. Miles S, Kellett J, Leinster SJ. Foundation doctors’ induction experiences. BMC Med Educ. 2015;15:118.

37. O’Donnell M, Noad R, Boohan M, Carragher A. Foundation Programme Impact on Junior Doctor Personality and Anxiety in Northern Ireland. Ulster Med J. :7.

38. Rogers E, Polonijo AN, Carpiano RM. Getting by with a little help from friends and colleagues: Testing how residents’ social support networks affect loneliness and burnout. Can Fam Physician Med Fam Can. 2016;62:e677–83.

39. Bruce C, Thomas PS, Yates DH. Health and stress in Australian interns: Health and stress in interns. Intern Med J. 2003;33:392–5.

40. McKavanagh P, Smyth A, Carragher A. Hospital consultants and workplace based assessments: how foundation doctors view these educational interactions? Postgrad Med J. 2012;88:119–24.

41. Shapiro RE, Vallejo MC, Sofka SH, Elmo RM, Anderson AH, Ferrari ND. Hospital Spiritual Care Can Complement Graduate Medical Trainee Well-Being. Adv Med. 2019;2019:1–4.

42. Arora VM, Greenstein EA, Woodruff JN, Staisiunas PG, Farnan JM. Implementing Peer Evaluation of Handoffs: Associations With Experience and Workload: Implementing Peer Evaluation of Handoffs. J Hosp Med. 2013;8:132–6.

43. Arora VM, Farnan JM, Lypson ML, Anderson RA, Prochaska MH, Humphrey HJ. Incoming Interns’ Perspectives on the Institute of Medicine Recommendations for Residents’ Duty Hours. J Grad Med Educ. 2010;2:536–40.

44. Gruppen LD, Stansfield RB, Zhao Z, Sen S. Institution and Specialty Contribute to Resident Satisfaction With Their Learning Environment and Workload: Acad Med. 2015;90:S77–82.

45. Røvik JO, Tyssen R, Hem E, Gude T, Ekeberg Ø, Moum T, et al. Job Stress in Young Physicians with an Emphasis on the Work-Home Interface: A Nine-Year, Nationwide and Longitudinal Study of its Course and Predictors. Ind Health. 2007;45:662–71.

46. Chandramouleeswaran S, Edwin NC, Braganza D. Job Stress, Satisfaction, and Coping Strategies Among Medical Interns in a South Indian Tertiary Hospital. Indian J Psychol Med. 2014;36:308–11.

47. Henning MA, Sollers J, Strom JM, Hill AG, Lyndon MP, Cumin D, et al. Junior doctors in their first year: mental health, quality of life, burnout and heart rate variability. Perspect Med Educ. 2014;3:136–43.

48. Byszewski A, Lochnan H, Johnston D, Seabrook C, Wood T. Learning environment: assessing resident experience. Clin Teach. 2017;14:195–9.

49. Keim SM, Mays MZ, Williams JM, Serido J, Harris RB. Measuring wellness among resident physicians. Med Teach. 2006;28:370–4.

50. Hoppe A, Persson E, Birgegård G. Medical interns’ view of their undergraduate medical education in Uppsala: An alumnus study with clear attitude differences between women and men. Med Teach. 2009;31:426–32.

51. Han E, Chung E, Oh S, Woo Y, Hitchcock M. Mentoring experience and its effects on medical interns. Singapore Med J. 2014;55:593–7.

52. Bu CNN, Cotzias E, Panagioti M. Mindfulness intervention for foundation year doctors: a feasibility study. Pilot Feasibility Stud. 2019;5:61.

53. Cave J, Goldacre M, Lambert T, Woolf K, Jones A, Dacre J. Newly qualified doctors’ views about whether their medical school had trained them well: questionnaire surveys. BMC Med Educ. 2007;7:38.

54. Kazmi R, Amjad S, Khan D. Occupational stress and its effect on job performance. A case study of medical house officers of district Abbottabad. J Ayub Med Coll Abbottabad JAMC. 2008;20:135–9.

55. Hassan M, Hussain T, Ahmed S, Fraz T, Rehmat Z. Perceived stress and stressors among house officers. Indian J Occup Environ Med. 2014;18:145.

56. Cedfeldt AS, Bower EA, English C, Grady-Weliky TA, Girard DE, Choi D. Personal time off and residents’ career satisfaction, attitudes and emotions: Time off and well-being in residents. Med Educ. 2010;44:977–84.

57. Kleim B, Thörn HA, Ehlert U. Positive interpretation bias predicts well-being in medical interns. Front Psychol. 2014;5:640.

58. Abuhusain H, Chotirmall SH, Hamid N, O’Neill SJ. Prepared for internship? Ir Med J. 2009;102:82–4.

59. Kelly C, Noonan CLF, Monagle JP. Preparedness for internship: a survey of new interns in a large Victorian Health Service. Aust Health Rev. 2011;35:146.

60. Goldacre MJ. Preregistration house officers’ views on whether their experience at medical school prepared them well for their jobs: national questionnaire survey. BMJ. 2003;326:1011–2.

61. Charalambous A, Pantelas G, Pouloukas S. Preregistration programme for medical practice: a survey of Cypriot trainees (2000 and 2002). East Mediterr Health J Rev Sante Mediterr Orient Al-Majallah Al-Sihhiyah Li-Sharq Al-Mutawassit. 2007;13:129–37.

62. Lau MW, Li WE, Llewellyn A, Cyna AM. Prevalence and associations of psychological distress in Australian junior medical officers: Psychological distress in JMO. Intern Med J. 2017;47:1190–6.

63. Lin Y-H, Lin S-H, Li P, Huang W-L, Chen C-Y. Prevalent hallucinations during medical internships: phantom vibration and ringing syndromes. PloS One. 2013;8:e65152.

64. Rodger M, Lavender T, Kapur N. Problem-based learning, work demands and psychological distress in pre-registration house officers: a preliminary study. Med Teach. 2002;24:334–6.

65. Al Sultan AI, Parashar SK, Wahass SH, Al Soweilem LS. Professional Stress During Medical Internship. Qatar Med J. 2002;2002:19.

66. Appelbaum NP, Santen SA, Aboff BM, Vega R, Munoz JL, Hemphill RR. Psychological Safety and Support: Assessing Resident Perceptions of the Clinical Learning Environment. J Grad Med Educ. 2018;10:651–6.

67. Newbury-Birch D. Psychological stress, anxiety, depression, job satisfaction, and personality characteristics in preregistration house officers. Postgrad Med J. 2001;77:109–11.

68. Choi D, Cedfeldt A, Flores C, Irish K, Brunett P, Girard D. Resident wellness: institutional trends over 10 years since 2003. Adv Med Educ Pract. 2017;Volume 8:513–23.

69. Reynolds PP, White C, Martindale JR. Residents’ perspective on professionalism in the learning environment. Curr Probl Pediatr Adolesc Health Care. 2019;49:84–91.

70. Holt KD, Miller RS, Philibert I, Heard JK, Nasca TJ. Residents’ perspectives on the learning environment: data from the Accreditation Council for Graduate Medical Education resident survey. Acad Med J Assoc Am Med Coll. 2010;85:512–8.

71. Baldwin DC, Daugherty SR, Ryan PM, Yaghmour NA, Philibert I. Residents’ Ratings of Their Clinical Supervision and Their Self-Reported Medical Errors: Analysis of Data From 2009. J Grad Med Educ. 2018;10:235–41.

72. Baldwin DC, Daugherty SR. Sleep Deprivation and Fatigue in Residency Training: Results of a National Survey of First- and Second-Year Residents. Sleep. 2004;27:217–23.

73. Min AA, Sbarra DA, Keim SM. Sleep disturbances predict prospective declines in resident physicians’ psychological well-being. Med Educ Online. 2015;20:28530.

74. Zebrowski JP, Pulliam SJ, Denninger JW, Berkowitz LR. So Tired: Predictive Utility of Baseline Sleep Screening in a Longitudinal Observational Survey Cohort of First-Year Residents. J Gen Intern Med. 2018;33:825–30.

75. Yusoff MSB, Jie TY, Esa AR. Stress, stressors and coping strategies among house officers in a Malaysian hospital. ASEAN J Psychiatry. Malaysia: Mobition Sdn. Bhd.; 2011;12:85–94.

76. Sangi-Haghpeykar H, Ambani DS, Carson SA. Stress, workload, sexual well-being and quality of life among physician residents in training. Int J Clin Pract. 2009;63:462–7.

77. Kashner TM, Henley SS, Golden RM, Byrne JM, Keitz SA, Cannon GW, et al. Studying the effects of ACGME duty hours limits on resident satisfaction: results from VA learners’ perceptions survey. Acad Med J Assoc Am Med Coll. 2010;85:1130–9.

78. Touchie C, De Champlain A, Pugh D, Downing S, Bordage G. Supervising incoming first-year residents: faculty expectations versus residents’ experiences. Med Educ. 2014;48:921–9.

79. Jagsi R. The Accreditation Council for Graduate Medical Education’s Limits on Residents’ Work Hours and Patient SafetyA Study of Resident Experiences and Perceptions Before and After Hours Reductions. Arch Intern Med. 2008;168:493.

80. Marek AP, Nygaard RM, Liang ET, Roetker NS, DeLaquil M, Gregorich S, et al. The association between objectively-measured activity, sleep, call responsibilities, and burnout in a resident cohort. BMC Med Educ. 2019;19:158.

81. Goldacre MJ, Davidson JM, Lambert TW. The first house officer year: views of graduate and non-graduate entrants to medical school. Med Educ. 2008;42:286–93.

82. Choi D, Dickey J, Wessel K, Girard DE. The impact of the implementation of work hour requirements on residents’ career satisfaction, attitudes and emotions. BMC Med Educ. 2006;6:53.

83. Degen C, Weigl M, Glaser J, Li J, Angerer P. The impact of training and working conditions on junior doctors’ intention to leave clinical practice. BMC Med Educ. 2014;14:119.

84. Burford B, Whittle V, Vance GH. The relationship between medical student learning opportunities and preparedness for practice: a questionnaire study. BMC Med Educ. 2014;14:223.

85. Anastasiadis C, Tsounis A, Sarafis P. The relationship between stress, social capital and quality of education among medical residents. BMC Res Notes. 2018;11:274.

86. Tyssen R, Vaglum P, Grønvold NT, Ekeberg Ø. The relative importance of individual and organizational factors for the prevention of job stress during internship: a nationwide and prospective study. Med Teach. 2005;27:726–31.

87. Bola S, Trollip E, Parkinson F. The state of South African internships: A national survey against HPCSA guidelines. S Afr Med J. 2015;105:535.

88. Doran T, Maudsley G, Zakhour H. Time to think? Questionnaire survey of pre-registration house officers’ experiences of critical appraisal in the Mersey Deanery. Med Educ. 2007;41:487–94.

89. Lambert TW, Surman G, Goldacre MJ. Views of UK-trained medical graduates of 1999–2009 about their first postgraduate year of training: national surveys. BMJ Open. 2013;3:e002723.

90. Guille C, Frank E, Zhao Z, Kalmbach DA, Nietert PJ, Mata DA, et al. Work-Family Conflict and the Sex Difference in Depression Among Training Physicians. JAMA Intern Med. 2017;177:1766.

91. Finucane P, O’Dowd T. Working and training as an intern: a national survey of Irish interns. Med Teach. 2005;27:107–13.

92. Swaid A, Elhilu A, Mahfouz M. Medical internship training in Saudi Arabia: interns’ views and perceptions. Adv Med Educ Pract. 2017;Volume 8:121–8.
