# Supplementary material for: Tools for measuring medical internship experience: a scoping review
Source: Hum Resour Health. 2021 Jan 14;19:10. doi: 10.1186/s12960-021-00554-7 (PMC7809831; doi:10.1186/s12960-021-00554-7)
Supplement: Supplementary file 4 — Additional file 4: Detailed information of tool and questionnaire used in 92 included studies [file 12960_2021_554_MOESM4_ESM.docx]

**Additional file 4. Detailed information of tool and questionnaire used in 92 included studies**

**Theme 1: Well-being**

| Tool name | Time used | Reference | Sub-themes | Internal validity evidence | Reliability evidence |
| --- | --- | --- | --- | --- | --- |
| Maslach Burnout Inventory (MBI) | 6 | (2,10,17–19,45) | Burnout | Previously tested | Previously tested |
| Patient Health Questionnaire-9 (PHQ-9) | 6 | (7,22,31,41,57,90) | Depression | Previously tested | (31) Internal consistency (Cronbach’s alpha = 0.74-0.85), test-retest |
| General Health Questionnaire-12 or 30 (GHQ-12 or GHQ-30) | 5 | (18,39,64,67,75) | Psychological distress | Previously tested | Previously tested |
| Perceived stress scale | 4 | (7,21,31,55) | Stress | Previously tested | (31) Internal consistency (Cronbach’s alpha = 0.82-90), test-retest |
| Hospital Anxiety and Depression Scale | 3 | (2,10,67) | Anxiety, depression | Previously tested | (10) Internal consistency (Cronbach’s alpha = 0.53) |
| Brief resident wellness profile | 2 | (49,73) | Wellness | (49) Convergent validity (r=0.63), discriminant validity (r=-0.37), concurrent validity (r=0.007), face validity | (73) Internal consistency (Cronbach’s alpha = 0.83) |
| Connor-Davidson-Resilience Scale | 2 | (7,57) | Stress | 0 | (57) Internal consistency (Cronbach’s alpha = 0.85) |
| Cooper Job Stress | 2 | (45,86) | Stress | (45) Construct validity (four factors), face validity  (86) Construct validity (three factors) | (45) Internal consistency (Cronbach’s alpha = 0.73-0.83)  (86) Internal consistency (Cronbach’s alpha = 0.90) |
| Copenhagen Burnout Inventory | 2 | (38,47) | Burnout | Previously tested | (38)Internal consistency (Cronbach’s alpha = 0.73-0.71 and 0.82) |
| Occupational Stress Indicator | 2 | (21,67) | Stress | 0 | (21) Internal consistency (Cronbach’s alpha = 0.89) |
| Pittsburgh Sleep Quality Index | 2 | (73,74) | Sleep | Previously tested | Previously tested |
| UK Medical Career Research Group | 2 | (26,89) | Job satisfaction | 0 | 0 |
| Anxiety about professional future | 1 | (12) | Anxiety | (12) Construct validity (one factor) | (12) Internal consistency (Cronbach’s alpha = 0.71) |
| Chalder Fatigue Scale | 1 | (34) | Fatigue | Previously tested | (34) Internal consistency (Cronbach’s alpha = 0.95) |
| Cohen Perceived Stress Scale | 1 | (34) | Stress | Previously tested | (34) Internal consistency (Cronbach’s alpha = 0.68) |
| Effort–Reward Imbalance (ERI) | 1 | (20) | Stress | Previously tested | (20) Internal consistency (Cronbach’s alpha = 0.70-82) |
| General stressor questionnaire | 1 | (75) | Stress | Previously tested | (75) Internal consistency (Cronbach’s alpha = 0.94) |
| Job satisfaction scale | 1 | (21) | Job satisfaction | Previously tested | (21) Internal consistency (Cronbach’s alpha = 0.89) |
| Medical Outcomes Study (MOS) Sleep Scale | 1 | (34) | Sleep | Previously tested | (34) Internal consistency (Cronbach’s alpha = 0.73) |
| Minnesota Satisfaction Questionnaire | 1 | (51) | Job satisfaction | Previously tested | (51) Internal consistency (Cronbach’s alpha = 0.941) |
| Positive and Negative Affect Schedule (PANAS) scales | 1 | (32) | Wellbeing | 0 | (32) Internal consistency (Cronbach’s alpha = 0.88-92) |
| State-Trait Anxiety Inventory | 1 | (37) | Anxiety | Previously tested | (37) Test-retest |
| State-Trait Depression Scale | 1 | (20) | Depression | Previously tested | (20) Internal consistency (Cronbach’s alpha = 0.88-90) |
| A modifield questionnaire by Buckey and Harasym | 1 | (65) | Stress | 0 | 0 |
| Arora et al. 2010 questionnaire | 1 | (43) | Fatigue | 0 | 0 |
| Baldwin & Daugherty. 2004 questionnaire | 1 | (72) | Stress, sleep | Previously tested | 0 |
| Beck depression and anxiety inventories | 1 | (63) | Depression, anxiety | Previously tested | Previously tested |
| Brief COPE | 1 | (75) | Stress | Previously tested | 0 |
| Bu et al. 2019 questionnaire | 1 | (52) | Stress, wellbeing | 0 | 0 |
| Burnout measure (BM) | 1 | (22) | Burnout | Previously tested | Previously tested |
| Cedfeldt et al. 2010 questionnaire | 1 | (56) | Stress, job satisfaction, wellbeing, sleep | Previously tested | 0 |
| Choi et al. 2006 questionnaire | 1 | (82) | Job satisfaction | Previously tested | 0 |
| Depression, Anxiety and Stress Scales | 1 | (47) | Depression, anxiety, stress | Previously tested | Previously tested |
| Epworth Sleepiness Scale | 1 | (74) | Sleep | Previously tested | Previously tested |
| Eurohis quality of life | 1 | (47) | Quality of life | Previously tested | Previously tested |
| Generalized anxiety disorder-7 (GAD-7) | 1 | (22) | Anxiety | Previously tested | Previously tested |
| Health Consultant’s Job Stress and Satisfaction questionnaire | 1 | (46) | Job satisfaction | 0 | 0 |
| Jagsi 2008 questionnaire | 1 | (79) | Fatigue | 0 | 0 |
| Jefferson Physician Empathy Scale | 1 | (2) | Empathy | Previously tested | 0 |
| Kalmbach et al. 2018 questionnaire | 1 | (30) | Sleep, mood | 0 | 0 |
| Kashner et al. 2010 questionnaire | 1 | (77) | Stress, fatigue | 0 | 0 |
| Kazmi et al. 2008 questionnaire | 1 | (54) | Stress | 0 | 0 |
| Kessler Psychological Distress Scale | 1 | (62) | Anxiety, depression | 0 | 0 |
| Marek et al. 2019 questionnaire | 1 | (80) | Burnout, sleep | Previously tested | 0 |
| Mataya et al. 2015 questionnaire | 1 | (33) | Job satisfaction, quality of life | 0 | 0 |
| Modified AMA survey | 1 | (5) | Stress, sleep, job satisfaction | 0 | 0 |
| Positive states of mind | 1 | (49) | Stress | Previously tested | Previously tested |
| Quality of life inventory | 1 | (76) | Quality of life | Previously tested | Previously tested |
| Satisfaction with Life Scale | 1 | (17) | Life satisfaction | Previously tested | Previously tested |
| Sources of Stress Scale (SSS) | 1 | (85) | Stress | 0 | 0 |
| Spielberger's Situational Anxiety Inventory | 1 | (29) | Anxiety | Previously tested | Previously tested |
| Stress inventory | 1 | (76) | Stress | 0 | 0 |
| Stress Profile | 1 | (49) | Stress | 0 | Previously tested |
| Swaid et al. 2017 questionnaire | 1 | (92) | Job satisfaction | 0 | 0 |

**Theme 2: Educational environment**

| Tool name | Time used | Reference | Sub-themes | Internal validity evidence | Reliability evidence | |
| --- | --- | --- | --- | --- | --- | --- |
| UK Medical Career Research Group survey | 5 | (26,53,60,81,89) | Support, handoff, induction, supervision, teaching, feedback, preparedness | 0 | 0 | |
| PHEEM | 4 | (15,24,35,85) | Educational environment, supervision, induction, communication, feedback, career development, teamwork, teaching, support | (24) Face validity, construct validity (three factors)  (15) construct validity (one factor) | (24) Internal consistency (Cronbach’s alpha = 0.93)  (15) Internal consistency (Cronbach’s alpha = 0.84) | |
| Accreditation Council for Graduate Medical Education Resident Survey | 2 | (16,70) | Teamwork, educational experience, handoff | 0 | 0 | |
| Junior Doctor Assessment Tool | 2 | (14,23) | Communication, teamwork, professionalism | (14) Face validity, construct validity (three factors) | (14) Internal consistency (Cronbach’s alpha = 0.883) | |
| Climate for learning | 1 | (86) | Learning environment, support | (86) Construct validity (two factors) | (86) Internal consistency (Cronbach’s alpha = 0.77) | |
| Cognitive Behaviour Survey - Residency | 1 | (6) | Learning environment | (6) Construct validity (seven factors) | (6) Internal consistency (Cronbach’s alpha = 0.85-89) | |
| Friesen et al. 2008 questionnaire | 1 | (34) | Teamwork | 0 | (34) Internal consistency (Cronbach’s alpha = 0.89) | |
| Graduate Medical Education Committee annual survey | 1 | [64] | Supervision, feedback | [64] Construct validity, content validity, discriminate validity | [64] Internal consistency (Cronbach’s alpha = 0.83-87) | |
| Handoff Clinical Evaluation Exercise | 1 | [65] | Professionalism, communication, feedback, handoff | Previously tested | [65] Internal consistency (Cronbach’s alpha = 0.98); Inter-rater reliability (ICC overall performance (0.18)) | |
| Hannan et al. 2017 questionnaire | 1 | [4] | Support, preparedness, teamwork | [4] Face validity | 0 | |
| Learning environment professionalism survey | 1 | [66] | Learning environment | Previously tested | [66] Internal consistency (Cronbach’s alpha = 0.78-88); test-retest reliability | |
| Lubben Social Network Scale | 1 | [22] | Support | 0 | [22] Internal consistency (Cronbach’s alpha = 0.75-84) | |
| Mentorship effectiveness Scale | 1 | [30] | Supervision | Previously tested | [30] Internal consistency (Cronbach’s alpha = 0.931) | |
| Modified Resident Questionnaire | 1 | [67] | Learning environment | 0 | [67] Internal consistency (Cronbach’s alpha = 0.84) | |
| Reynolds et al. 2019 questionnaire | 1 | [68] | Professionalism | 0 | [68] Internal consistency (Cronbach’s alpha unknown) | |
| Safety Attitudes Questionnaire | 1 | [69] | Teamwork, professionalism | [69] Concurrent validity | Previously tested | |
| Short Survey of Perceived Organizational Support | 1 | [70] | Support, learning environment | [70] Construct validity, concurrent validity | [70] Internal consistency (Cronbach’s alpha = 0.94) | |
| Speaking Up Climates | 1 | [69] | Teamwork, professionalism | [69] Face validity, concurrent validity, discriminate validity, construct validity (one factor) | [69] Internal consistency (Cronbach’s alpha > 0.70) | |
| Touchie et al. 2014 questionnaire | 1 | [71] | Supervision | [71] Face validity | 0 | |
| Work Analysis Instrument for Hospitals | 1 | [72] | Teaching, support | Previously tested | [72] Internal consistency (Cronbach’s alpha = 0.64-87) | |
| Yusoff et al. 2011 questionnaire | 1 | [16] | Teamwork, supervision, support | Previously tested | [16] Internal consistency (Cronbach’s alpha for individual tools including GSQ) | |
| A 60-item instrument developed by Bellini | 1 | [73] | Feedback, learning environment, supervision | 0 | 0 | |
| Abuhusain et al. 2009 questionnaire | 1 | [74] | Preparedness, induction, learning environment | 0 | 0 | |
| Arora et al. 2010 questionnaire | 1 | [34] | Supervision | 0 | 0 | |
| Baldwin & Daugherty 2004 questionnaire | 1 | [35] | Supervision, overall educational experience, professionalism | Previously tested | 0 |  |
| Baldwin et al. 2018 questionnaire | 1 | [75] | Supervision | 0 | 0 | |
| Bola et al. 2015 questionnaire | 1 | [76] | Supervision, teaching, handoff, induction | Previously tested | 0 | |
| Burford et al. 2014 questionnaire | 1 | [77] | Preparedness, overall educational experience | 0 | 0 | |
| Chu et al. 2009 questionnaire | 1 | [78] | Handoff, supervision | 0 | 0 | |
| Copenhagen Psychosocial Questionnaire | 1 | [3] | Support | Previously tested | Previously tested | |
| Doran et al. 2007 questionnaire | 1 | [79] | Preparedness, supervision | 0 | 0 | |
| Finucane & O’Dowd 2005 questionnaire | 1 | [80] | Teaching, feedback, preparedness | 0 | 0 | |
| Han & Maxwell 2006 questionnaire | 1 | [81] | Preparedness | 0 | 0 | |
| Hoppe et al. 2009 questionnaire | 1 | [82] | Preparedness, feedback, supervision, communication, training | Previously tested | 0 | |
| Kashner et al. 2010 questionnaire | 1 | [43] | Supervision, learning environment, support, teamwork, teaching | 0 | 0 | |
| Kazmi et al. 2008 questionnaire | 1 | [44] | Communication, supervision | 0 | 0 |  |
| Kelly et al. 2011 questionnaire | 1 | [83] | Preparedness | 0 | 0 | |
| Linklater 2010 questionnaire | 1 | [84] | Preparedness, support | 0 | 0 | |
| Mataya et al. 2015 questionnaire | 1 | [47] | Learning environment, supervision, career development | 0 | 0 | |
| McKavanagh et al. 2012 questionnaire | 1 | [85] | Supervision, feedback | 0 | 0 | |
| Miles et al. 2015 questionnaire | 1 | [86] | Induction | 0 | 0 | |
| Modified AMA survey | 1 | [48] | Supervision, professionalism | 0 | 0 | |
| Modified Manchester Questionnaire | 1 | [87] | Preparedness | 0 | 0 | |
| Modified questionnaire by Buckey and Harasym | 1 | [33] | Supervision, feedback, communication | 0 | 0 | |
| Operating Team Resource Management Survey | 1 | [88] | Teamwork, professionalism | 0 | 0 | |
| Rodger et al. 2002 questionnaire | 1 | [14] | Support, supervision | 0 | 0 | |
| Ross et al. 2018 questionnaire | 1 | [89] | Supervision, teaching | 0 | 0 | |
| Sarason Social Support Questionnaire | 1 | [7] | Support | 0 | 0 |  |
| Swaid et al. 2017 questionnaire | 1 | [52] | Induction, preparedness, teaching | 0 | 0 | |

**Theme 3: Work condition and environment**

| Tool name | Time used | Reference | Sub-themes | Internal validity evidence | Reliability evidence | |
| --- | --- | --- | --- | --- | --- | --- |
| PHEEM | 4 | [49,56–58] | Work hour, harassment or bullying, safety, food and accommodation | [57] Face validity, construct validity (three factors)  [56] construct validity (one factor) | [57] Internal consistency (Cronbach’s alpha = 0.93)  [56] Internal consistency (Cronbach’s alpha = 0.84) | |
| UK Medical Career Research Group survey | 3 | [25,26,55] | Workload, food and accommodation, remuneration | 0 | 0 | |
| Accreditation Council for Graduate Medical Education Resident Survey | 2 | [59,60] | Workload, safety | 0 | 0 | |
| Cyber Negative Acts Questionnaire | 1 | [31] | Bullying | Previously tested | [31] Internal consistency (Cronbach’s alpha = 0.85) | |
| Graduate Medical Education Committee annual survey | 1 | [64] | Workload | [64] Construct validity, content validity, discriminate validity | [64] Internal consistency (Cronbach’s alpha = 0.83-87) | |
| Hannan et al. 2017 questionnaire | 1 | [4] | Workload | [4] Face validity | 0 | |
| Modified Resident Questionnaire | 1 | [67] | Workload | 0 | [67] Internal consistency (Cronbach’s alpha = 0.85) | |
| Psychological Safety Scale | 1 | [70] | Safety | [70] Concurrent validity | [70] Internal consistency (Cronbach’s alpha = 0.76) | |
| Robson et al. 2011 questionnaire | 1 | [90] | Safety | [90] Content validity (0.88), construct validity | 0 | |
| Safety Attitudes Questionnaire | 1 | [69] | Safety | [69] Concurrent validity | Previously tested | |
| Speaking Up Climates | 1 | [69] | Safety | [69] Face validity, concurrent validity, discriminate validity, construct validity (one factor) | [69] Internal consistency (Cronbach’s alpha > 0.70) | |
| Work Analysis Instrument for Hospitals | 1 | [72] | Workload | Previously tested | [72] Internal consistency (Cronbach’s alpha = 0.64-87) | |
| Yusoff et al. 2011 questionnaire | 1 | [16] | Workload, pay and remuneration, work-family conflict | Previously tested | [16] Internal consistency (Cronbach’s alpha for individual tools including GSQ) | |
| A 60-item instrument developed by Bellini | 1 | [73] | Workload, infrastructure, harassment, pay and renumeration, food and accommodation | 0 | 0 | |
| Arora et al. 2010 questionnaire | 1 | [34] | Workload | 0 | 0 | |
| Baldwin & Daugherty 2004 questionnaire | 1 | [35] | Work hours, harassment | Previously tested | 0 | |
| Bola et al. 2015 questionnaire | 1 | [76] | Workload, safety | Previously tested | 0 |  |
| Choi et al. 2006 questionnaire | 1 | [39] | Work hours | 0 | 0 | |
| Choi et al. 2017 questionnaire | 1 | [91] | Work hours | 0 | 0 | |
| Doran et al. 2007 questionnaire | 1 | [79] | Workload | 0 | 0 |  |
| Finucane & O’Dowd 2005 questionnaire | 1 | [80] | Accommodation, pay, harassment, workload | 0 | 0 |  |
| Friesen et al. 2008 questionnaire | 1 | [28] | Work hours | 0 | 0 | |
| Hinze 2004 questionnaire | 1 | [92] | Harassment | 0 | 0 | |
| Jagsi et al. 2008 questionnaire | 1 | [41] | Work hours, safety | 0 | 0 | |
| Kashner et al. 2010 questionnaire | 1 | [43] | Work hours, infrastructure, safety, food and accommodation | 0 | 0 | |
| Kazmi et al. 2008 questionnaire | 1 | [44] | Workload | 0 | 0 | |
| Keim et al. 2006 questionnaire | 1 | [19] | Work hours | 0 | 0 | |
| Lau et al. 2017 questionnaire | 1 | [45] | Work hours | 0 | 0 | |
| Mataya et al. 2015 questionnaire | 1 | [47] | Work hours, pay and remuneration | 0 | 0 | |
| Mayer 2017 questionnaire | 1 | [9] | Work hours | 0 | 0 | |
| Modified AMA survey | 1 | [48] | Work hour, harassment | 0 | 0 | |
| Modified questionnaire by Buckey and Harasym | 1 | [33] | Work hours, food and accommodation, harassment or bullying | 0 | 0 | |
| Operating Team Resource Management Survey | 1 | [88] | Safety | 0 | 0 | |
| Rodger et al. 2002 questionnaire | 1 | [14] | Workload | 0 | 0 | |
| Rovik et al. 2007 questionnaire | 1 | [6] | Work hours | 0 | 0 | |
| Sen et al. 2010 questionnaire | 1 | [7] | Work hours | 0 | 0 | |
| Ross et al. 2018 questionnaire | 1 | [89] | Work hours, infrastructure | 0 | 0 | |
| Work and Family Conflict Scale | 1 | [12] | Work-family conflict | Previously tested | Previously tested | |

**Reference**

1. Galam E, Vauloup Soupault C, Bunge L, Buffel du Vaure C, Boujut E, Jaury P. ‘Intern life’: a longitudinal study of burnout, empathy, and coping strategies used by French GPs in training. BJGP Open. 2017;1:bjgpopen17X100773.

2. Brant H, Wetherell MA, Lightman S, Crown A, Vedhara K. An exploration into physiological and self-report measures of stress in pre-registration doctors at the beginning and end of a clinical rotation. Stress. 2010;13:155–62.

3. Lin KS, Zaw T, Oo WM, Soe PP. Burnout among house officers in Myanmar: A cross-sectional study. Ann Med Surg. 2018;33:7–12.

4. Hannan E, Breslin N, Doherty E, McGreal M, Moneley D, Offiah G. Burnout and stress amongst interns in Irish hospitals: contributing factors and potential solutions. Ir J Med Sci 1971 -. 2018;187:301–7.

5. Calcides DAP, Didou R da N, Melo EV de, Oliva-Costa EF de. Burnout Syndrome in medical internship students and its prevention with Balint Group. Rev Assoc Médica Bras. 2019;65:1362–7.

6. Røvik JO, Tyssen R, Hem E, Gude T, Ekeberg Ø, Moum T, et al. Job Stress in Young Physicians with an Emphasis on the Work-Home Interface: A Nine-Year, Nationwide and Longitudinal Study of its Course and Predictors. Ind Health. 2007;45:662–71.

7. Sen S, Kranzler HR, Krystal JH, Speller H, Chan G, Gelernter J, et al. A Prospective Cohort Study Investigating Factors Associated With Depression During Medical Internship. Arch Gen Psychiatry. 2010;67:557.

8. Talih F, Warakian R, Ajaltouni J, Shehab AAS, Tamim H. Correlates of Depression and Burnout Among Residents in a Lebanese Academic Medical Center: a Cross-Sectional Study. Acad Psychiatry. 2016;40:38–45.

9. Mayer S. Examining the Relationships Between Chronic Stress, HPA Axis Activity, and Depression in a Prospective and Longitudinal Study of Medical Internship [Internet]. University of Michigan; 2017. Available from: https://deepblue.lib.umich.edu/handle/2027.42/137074

10. Shapiro RE, Vallejo MC, Sofka SH, Elmo RM, Anderson AH, Ferrari ND. Hospital Spiritual Care Can Complement Graduate Medical Trainee Well-Being. Adv Med. 2019;2019:1–4.

11. Kleim B, Thörn HA, Ehlert U. Positive interpretation bias predicts well-being in medical interns. Front Psychol. 2014;5:640.

12. Guille C, Frank E, Zhao Z, Kalmbach DA, Nietert PJ, Mata DA, et al. Work-Family Conflict and the Sex Difference in Depression Among Training Physicians. JAMA Intern Med. 2017;177:1766.

13. Bruce C, Thomas PS, Yates DH. Health and stress in Australian interns: Health and stress in interns. Intern Med J. 2003;33:392–5.

14. Rodger M, Lavender T, Kapur N. Problem-based learning, work demands and psychological distress in pre-registration house officers: a preliminary study. Med Teach. 2002;24:334–6.

15. Newbury-Birch D. Psychological stress, anxiety, depression, job satisfaction, and personality characteristics in preregistration house officers. Postgrad Med J. 2001;77:109–11.

16. Yusoff MSB, Jie TY, Esa AR. Stress, stressors and coping strategies among house officers in a Malaysian hospital. ASEAN J Psychiatry. Malaysia: Mobition Sdn. Bhd.; 2011;12:85–94.

17. Vinothkumar M, Arathi A, Joseph M, Nayana P, Jishma Ej, Sahana U. Coping, perceived stress, and job satisfaction among medical interns: The mediating effect of mindfulness. Ind Psychiatry J. 2016;25:195.

18. Hassan M, Hussain T, Ahmed S, Fraz T, Rehmat Z. Perceived stress and stressors among house officers. Indian J Occup Environ Med. 2014;18:145.

19. Keim SM, Mays MZ, Williams JM, Serido J, Harris RB. Measuring wellness among resident physicians. Med Teach. 2006;28:370–4.

20. Min AA, Sbarra DA, Keim SM. Sleep disturbances predict prospective declines in resident physicians’ psychological well-being. Med Educ Online. 2015;20:28530.

21. Tyssen R, Vaglum P, Grønvold NT, Ekeberg Ø. The relative importance of individual and organizational factors for the prevention of job stress during internship: a nationwide and prospective study. Med Teach. 2005;27:726–31.

22. Rogers E, Polonijo AN, Carpiano RM. Getting by with a little help from friends and colleagues: Testing how residents’ social support networks affect loneliness and burnout. Can Fam Physician Med Fam Can. 2016;62:e677–83.

23. Henning MA, Sollers J, Strom JM, Hill AG, Lyndon MP, Cumin D, et al. Junior doctors in their first year: mental health, quality of life, burnout and heart rate variability. Perspect Med Educ. 2014;3:136–43.

24. Zebrowski JP, Pulliam SJ, Denninger JW, Berkowitz LR. So Tired: Predictive Utility of Baseline Sleep Screening in a Longitudinal Observational Survey Cohort of First-Year Residents. J Gen Intern Med. 2018;33:825–30.

25. Goldacre MJ, Davidson JM, Lambert TW. Doctors’ views of their first year of medical work and postgraduate training in the UK: questionnaire surveys. Med Educ. 2003;37:802–8.

26. Lambert TW, Surman G, Goldacre MJ. Views of UK-trained medical graduates of 1999–2009 about their first postgraduate year of training: national surveys. BMJ Open. 2013;3:e002723.

27. Bolanowski W. Anxiety about professional future among young doctors. Int J Occup Med Environ Health. 2005;18:367–74.

28. Friesen LD, Vidyarthi AR, Baron RB, Katz PP. Factors associated with intern fatigue. J Gen Intern Med. 2008;23:1981–6.

29. Li J, Weigl M, Glaser J, Petru R, Siegrist J, Angerer P. Changes in psychosocial work environment and depressive symptoms: A prospective study in junior physicians: Changes in ERI and Depressive Symptoms. Am J Ind Med. 2013;56:1414–22.

30. Han E, Chung E, Oh S, Woo Y, Hitchcock M. Mentoring experience and its effects on medical interns. Singapore Med J. 2014;55:593–7.

31. Farley S, Coyne I, Sprigg C, Axtell C, Subramanian G. Exploring the impact of workplace cyberbullying on trainee doctors. Med Educ. 2015;49:436–43.

32. O’Donnell M, Noad R, Boohan M, Carragher A. Foundation Programme Impact on Junior Doctor Personality and Anxiety in Northern Ireland. Ulster Med J. :7.

33. Al Sultan AI, Parashar SK, Wahass SH, Al Soweilem LS. Professional Stress During Medical Internship. Qatar Med J. 2002;2002:19.

34. Arora VM, Farnan JM, Lypson ML, Anderson RA, Prochaska MH, Humphrey HJ. Incoming Interns’ Perspectives on the Institute of Medicine Recommendations for Residents’ Duty Hours. J Grad Med Educ. 2010;2:536–40.

35. Baldwin DC, Daugherty SR. Sleep Deprivation and Fatigue in Residency Training: Results of a National Survey of First- and Second-Year Residents. Sleep. 2004;27:217–23.

36. Lin Y-H, Lin S-H, Li P, Huang W-L, Chen C-Y. Prevalent hallucinations during medical internships: phantom vibration and ringing syndromes. PloS One. 2013;8:e65152.

37. Bu CNN, Cotzias E, Panagioti M. Mindfulness intervention for foundation year doctors: a feasibility study. Pilot Feasibility Stud. 2019;5:61.

38. Cedfeldt AS, Bower EA, English C, Grady-Weliky TA, Girard DE, Choi D. Personal time off and residents’ career satisfaction, attitudes and emotions: Time off and well-being in residents. Med Educ. 2010;44:977–84.

39. Choi D, Dickey J, Wessel K, Girard DE. The impact of the implementation of work hour requirements on residents’ career satisfaction, attitudes and emotions. BMC Med Educ. 2006;6:53.

40. Chandramouleeswaran S, Edwin NC, Braganza D. Job Stress, Satisfaction, and Coping Strategies Among Medical Interns in a South Indian Tertiary Hospital. Indian J Psychol Med. 2014;36:308–11.

41. Jagsi R. The Accreditation Council for Graduate Medical Education’s Limits on Residents’ Work Hours and Patient SafetyA Study of Resident Experiences and Perceptions Before and After Hours Reductions. Arch Intern Med. 2008;168:493.

42. Kalmbach DA, Fang Y, Arnedt JT, Cochran AL, Deldin PJ, Kaplin AI, et al. Effects of Sleep, Physical Activity, and Shift Work on Daily Mood: a Prospective Mobile Monitoring Study of Medical Interns. J Gen Intern Med. 2018;33:914–20.

43. Kashner TM, Henley SS, Golden RM, Byrne JM, Keitz SA, Cannon GW, et al. Studying the effects of ACGME duty hours limits on resident satisfaction: results from VA learners’ perceptions survey. Acad Med J Assoc Am Med Coll. 2010;85:1130–9.

44. Kazmi R, Amjad S, Khan D. Occupational stress and its effect on job performance. A case study of medical house officers of district Abbottabad. J Ayub Med Coll Abbottabad JAMC. 2008;20:135–9.

45. Lau MW, Li WE, Llewellyn A, Cyna AM. Prevalence and associations of psychological distress in Australian junior medical officers: Psychological distress in JMO. Intern Med J. 2017;47:1190–6.

46. Marek AP, Nygaard RM, Liang ET, Roetker NS, DeLaquil M, Gregorich S, et al. The association between objectively-measured activity, sleep, call responsibilities, and burnout in a resident cohort. BMC Med Educ. 2019;19:158.

47. Mataya AA, Macuvele ME, Gwitima T, Muula AS. Factors affecting job satisfaction and commitment among medical interns in Malawi: a cross-sectional study. Pan Afr Med J [Internet]. 2015 [cited 2020 Sep 9];21. Available from: http://www.panafrican-med-journal.com/content/article/21/174/full/

48. Baldwin DC, Daugherty SR, Tsai R, Scotti MJ. A national survey of residents’ self-reported work hours: thinking beyond specialty. Acad Med J Assoc Am Med Coll. 2003;78:1154–63.

49. Anastasiadis C, Tsounis A, Sarafis P. The relationship between stress, social capital and quality of education among medical residents. BMC Res Notes. 2018;11:274.

50. Mohammadi G, Tourdeh M, Ebrahimian A. Effect of simulation-based training method on the psychological health promotion in operating room students during the educational internship. J Educ Health Promot. 2019;8:172.

51. Sangi-Haghpeykar H, Ambani DS, Carson SA. Stress, workload, sexual well-being and quality of life among physician residents in training. Int J Clin Pract. 2009;63:462–7.

52. Swaid A, Elhilu A, Mahfouz M. Medical internship training in Saudi Arabia: interns’ views and perceptions. Adv Med Educ Pract. 2017;Volume 8:121–8.

53. Cave J, Goldacre M, Lambert T, Woolf K, Jones A, Dacre J. Newly qualified doctors’ views about whether their medical school had trained them well: questionnaire surveys. BMC Med Educ. 2007;7:38.

54. Goldacre MJ. Preregistration house officers’ views on whether their experience at medical school prepared them well for their jobs: national questionnaire survey. BMJ. 2003;326:1011–2.

55. Goldacre MJ, Davidson JM, Lambert TW. The first house officer year: views of graduate and non-graduate entrants to medical school. Med Educ. 2008;42:286–93.

56. Gooneratne IK, Munasinghe SR, Siriwardena C, Olupeliyawa AM, Karunathilake I. Assessment of psychometric properties of a modified PHEEM questionnaire. Ann Acad Med Singapore. 2008;37:993–7.

57. Roff S, McAleer S, Skinner A. Development and validation of an instrument to measure the postgraduate clinical learning and teaching educational environment for hospital-based junior doctors in the UK. Med Teach. 2005;27:326–31.

58. Auret K, Skinner L, Sinclair C, Evans S. Formal assessment of the educational environment experienced by interns placed in rural hospitals in Western Australia. :12.

59. Loftus TJ, Hall DJ, Malaty JZ, Kuruppacherry SB, Sarosi GA, Shaw CM, et al. Associations Between National Board Exam Performance and Residency Program Emphasis on Patient Safety and Interprofessional Teamwork. Acad Psychiatry. 2019;43:581–4.

60. Holt KD, Miller RS, Philibert I, Heard JK, Nasca TJ. Residents’ perspectives on the learning environment: data from the Accreditation Council for Graduate Medical Education resident survey. Acad Med J Assoc Am Med Coll. 2010;85:512–8.

61. Carr SE, Celenza A, Lake F. Assessment of Junior Doctor performance: a validation study. BMC Med Educ. 2013;13:129.

62. Carr SE, Celenza T, Lake FR. Descriptive analysis of junior doctor assessment in the first postgraduate year. Med Teach. 2014;36:983–90.

63. Mitchell R, Regan-Smith M, Fisher MA, Knox I, Lambert DR. A New Measure of the Cognitive, Metacognitive, and Experiential Aspects of Residents’ Learning: Acad Med. 2009;84:918–26.

64. Heard JK, O’Sullivan P, Smith CE, Harper RA, Schexnayder SM. An Institutional System to Monitor and Improve the Quality of Residency Education: Acad Med. 2004;79:858–64.

65. Arora VM, Greenstein EA, Woodruff JN, Staisiunas PG, Farnan JM. Implementing Peer Evaluation of Handoffs: Associations With Experience and Workload: Implementing Peer Evaluation of Handoffs. J Hosp Med. 2013;8:132–6.

66. Byszewski A, Lochnan H, Johnston D, Seabrook C, Wood T. Learning environment: assessing resident experience. Clin Teach. 2017;14:195–9.

67. Gruppen LD, Stansfield RB, Zhao Z, Sen S. Institution and Specialty Contribute to Resident Satisfaction With Their Learning Environment and Workload: Acad Med. 2015;90:S77–82.

68. Reynolds PP, White C, Martindale JR. Residents’ perspective on professionalism in the learning environment. Curr Probl Pediatr Adolesc Health Care. 2019;49:84–91.

69. Martinez W, Etchegaray JM, Thomas EJ, Hickson GB, Lehmann LS, Schleyer AM, et al. ‘Speaking up’ about patient safety concerns and unprofessional behaviour among residents: validation of two scales. BMJ Qual Saf. 2015;24:671–80.

70. Appelbaum NP, Santen SA, Aboff BM, Vega R, Munoz JL, Hemphill RR. Psychological Safety and Support: Assessing Resident Perceptions of the Clinical Learning Environment. J Grad Med Educ. 2018;10:651–6.

71. Touchie C, De Champlain A, Pugh D, Downing S, Bordage G. Supervising incoming first-year residents: faculty expectations versus residents’ experiences. Med Educ. 2014;48:921–9.

72. Degen C, Weigl M, Glaser J, Li J, Angerer P. The impact of training and working conditions on junior doctors’ intention to leave clinical practice. BMC Med Educ. 2014;14:119.

73. Charalambous A, Pantelas G, Pouloukas S. Preregistration programme for medical practice: a survey of Cypriot trainees (2000 and 2002). East Mediterr Health J Rev Sante Mediterr Orient Al-Majallah Al-Sihhiyah Li-Sharq Al-Mutawassit. 2007;13:129–37.

74. Abuhusain H, Chotirmall SH, Hamid N, O’Neill SJ. Prepared for internship? Ir Med J. 2009;102:82–4.

75. Baldwin DC, Daugherty SR, Ryan PM, Yaghmour NA, Philibert I. Residents’ Ratings of Their Clinical Supervision and Their Self-Reported Medical Errors: Analysis of Data From 2009. J Grad Med Educ. 2018;10:235–41.

76. Bola S, Trollip E, Parkinson F. The state of South African internships: A national survey against HPCSA guidelines. S Afr Med J. 2015;105:535.

77. Burford B, Whittle V, Vance GH. The relationship between medical student learning opportunities and preparedness for practice: a questionnaire study. BMC Med Educ. 2014;14:223.

78. Chu ES, Reid M, Schulz T, Burden M, Mancini D, Ambardekar AV, et al. A Structured Handoff Program for Interns: Acad Med. 2009;84:347–52.

79. Doran T, Maudsley G, Zakhour H. Time to think? Questionnaire survey of pre-registration house officers’ experiences of critical appraisal in the Mersey Deanery. Med Educ. 2007;41:487–94.

80. Finucane P, O’Dowd T. Working and training as an intern: a national survey of Irish interns. Med Teach. 2005;27:107–13.

81. Han W, Maxwell S. Are Medical Students Adequately Trained to Prescribe at the Point of Graduation? Views of First Year Foundation Doctors. Scott Med J. 2006;51:27–32.

82. Hoppe A, Persson E, Birgegård G. Medical interns’ view of their undergraduate medical education in Uppsala: An alumnus study with clear attitude differences between women and men. Med Teach. 2009;31:426–32.

83. Kelly C, Noonan CLF, Monagle JP. Preparedness for internship: a survey of new interns in a large Victorian Health Service. Aust Health Rev. 2011;35:146.

84. Linklater G. Educational needs of foundation doctors caring for dying patients. J R Coll Physicians Edinb. 2010;40:13–8.

85. McKavanagh P, Smyth A, Carragher A. Hospital consultants and workplace based assessments: how foundation doctors view these educational interactions? Postgrad Med J. 2012;88:119–24.

86. Miles S, Kellett J, Leinster SJ. Foundation doctors’ induction experiences. BMC Med Educ. 2015;15:118.

87. Bleakley A, Brennan N. Does undergraduate curriculum design make a difference to readiness to practice as a junior doctor? Med Teach. 2011;33:459–67.

88. Kobayashi H. A cross-cultural survey of residents’ perceived barriers in questioning/challenging authority. Qual Saf Health Care. 2006;15:277–83.

89. Ross A, Naidoo S (Cyril), Dlamini S. An evaluation of the medical internship programme at King Edward VIII hospital, South Africa in 2016. South Afr Fam Pract. 2018;60:187–91.

90. Robson J, de Wet C, McKay J, Bowie P. Do we know what foundation year doctors think about patient safety incident reporting? Development of a web based tool to assess attitude and knowledge. Postgrad Med J. 2011;87:750–6.

91. Choi D, Cedfeldt A, Flores C, Irish K, Brunett P, Girard D. Resident wellness: institutional trends over 10 years since 2003. Adv Med Educ Pract. 2017;Volume 8:513–23.

92. Hinze SW. ‘Am I Being Over-Sensitive?’ Women’s Experience of Sexual Harassment During Medical Training. Health Interdiscip J Soc Study Health Illn Med. 2004;8:101–27.
